# Evaluation of polydentate picolinic acid chelating ligands and an α-melanocyte-stimulating hormone derivative for targeted alpha therapy using ISOL-produced ^225^Ac

**DOI:** 10.1186/s41181-019-0072-5

**Published:** 2019-08-06

**Authors:** Caterina F. Ramogida, Andrew K. H. Robertson, Una Jermilova, Chengcheng Zhang, Hua Yang, Peter Kunz, Jens Lassen, Ivica Bratanovic, Victoria Brown, Lily Southcott, Cristina Rodríguez-Rodríguez, Valery Radchenko, François Bénard, Chris Orvig, Paul Schaffer

**Affiliations:** 1 Life Sciences Division, TRIUMF, 4004 Wesbrook Mall, Vancouver, BC V6T 2A3 Canada; 2 Department of Chemistry, Simon Fraser University, 8888 University Dr, Burnaby, BC V5A 1S6 Canada; 3 Department of Physics & Astronomy, University of British Columbia, 6224 Agricultural Road, Vancouver, BC V6T 1Z1 Canada; 4 Department of Molecular Oncology, BC Cancer Research Centre, 675 West 10th Ave, Vancouver, BC V5Z 1L3 Canada; 5 Accelerator Division, TRIUMF, 4004 Wesbrook Mall, Vancouver, BC V6T 2A3 Canada; 6 Faculty of Pharmaceutical Sciences, University of British Columbia, 2405 Wesbrook Mall, Vancouver, BC V6T 1Z3 Canada; 7 Department of Functional Imaging, BC Cancer, 600 West 10th Ave, Vancouver, BC V5Z 4E6 Canada; 8 Department of Radiology, University of British Columbia, 2775 Laurel St, Vancouver, BC V5Z 1M9 Canada; 9 Medicinal Inorganic Chemistry Group, Department of Chemistry, University of British Columbia, 2036 Main Mall, Vancouver, BC V6T 1Z1 Canada

**Keywords:** Actinium-225, ^225^Ac, Targeted alpha therapy, Isotope separation on-line, ISAC ISOL, Radionuclide production, Chelating ligands, Radiolabeling, α-Melanocyte-stimulating hormone

## Abstract

**Background:**

Actinium-225 (^225^Ac, t_1/2_ = 9.9 d) is a promising candidate radionuclide for use in targeted alpha therapy (TAT), though the currently limited global supply has hindered the development of a suitable Ac-chelating ligand and ^225^Ac-radiopharmaceuticals towards the clinic. We at TRIUMF have leveraged our Isotope Separation On-Line (ISOL) facility to produce ^225^Ac and use the resulting radioactivity to screen a number of potential ^225^Ac-radiopharmaceutical compounds.

**Results:**

MBq quantities of ^225^Ac and parent radium-225 (^225^Ra, t_1/2_ = 14.8 d) were produced and separated using solid phase extraction DGA resin, resulting in a radiochemically pure ^225^Ac product in > 98% yield and in an amenable form for radiolabeling of ligands and bioconjugates. Of the many polydentate picolinic acid (“pa”) containing ligands evaluated (H_4_octapa [N_4_O_4_], H_4_*CHX*octapa [N_4_O_4_], *p-*NO_2_-Bn-H_4_neunpa [N_5_O_4_], and H_6_phospa [N_4_O_4_]), all out-performed the current gold standard, DOTA for ^225^Ac radiolabeling ability at ambient temperature. Moreover, a melanocortin 1 receptor-targeting peptide conjugate, DOTA-modified cyclized α-melanocyte-stimulating hormone (DOTA-CycMSH), was radiolabeled with ^225^Ac and proof-of-principle biodistribution studies using B16F10 tumour-bearing mice were conducted. At 2 h post-injection, tumour-to-blood ratios of 20.4 ± 3.4 and 4.8 ± 2.4 were obtained for the non-blocking (molar activity [M.A.] > 200 kBq/nmol) and blocking (M.A. = 1.6 kBq/nmol) experiment, respectively.

**Conclusion:**

TRIUMF’s ISOL facility is able to provide ^225^Ac suitable for preclinical screening of radiopharmaceutical compounds; [^225^Ac(octapa)]^−^, [^225^Ac(*CHX*octapa)]^−^, and [^225^Ac(DOTA-CycMSH)] may be good candidates for further targeted alpha therapy studies.

**Electronic supplementary material:**

The online version of this article (10.1186/s41181-019-0072-5) contains supplementary material, which is available to authorized users.

## Background

Due to recent clinical results demonstrating the exceptional ability of ^225^Ac-radiopharmaceuticals for the treatment of late stage castration resistant prostate cancer (Sathekge et al. 2018; Kratochwil et al. 2016; Kratochwil et al. 2018), considerable efforts within the field of nuclear medicine have been directed towards development of new radiopharmaceuticals for targeted alpha therapy (TAT) containing ^225^Ac or other suitable alpha-emitting radionuclides (Poty et al. 2018a; Poty et al. 2018b; Seidl 2014; Kim and Brechbiel 2012; 11th International Symposium on Targeted Alpha Therapy (TAT11), 2019; Elgqvist et al. 2014; Morgenstern et al. 2018; Baidoo et al. 2013). Despite these promising preliminary findings, the progression of ^225^Ac drugs towards the clinic has been obstructed by the limited radionuclide supply and the limited investigation of radiochemical protocols for chelating ^225^Ac^III^ under mild conditions. Currently, the total global supply of ^225^Ac is approximately 63 GBq (1.7 Ci) per year, slowing clinical trials and resulting in high costs that make ^225^Ac inaccessible to many researchers. The reader is referred to the following reviews for a more detailed overview of ^225^Ac supply (Robertson et al. 2018; Engle 2018).

Moreover, the absence of a stable surrogate isotope makes the study of actinium chemistry particularly challenging, and only very recently have insights into the fundamental chemical properties and coordination preferences of the actinide appeared in the literature (Ferrier et al. 2017; Ferrier et al. 2016). The tetraaza macrocycle DOTA (1,4,7,10-tetraazacyclododecane-1,4,7,10-tetraacetic acid, N_4_O_4_), is currently the “gold standard” in ^225^Ac chelation, despite the chelating ligand’s sub-optimal properties for encapsulating this large actinide. DOTA forms kinetically inert complexes with ^225^Ac, albeit at a cost of slow radiolabeling kinetics which requires heating to elevated temperatures and extended reaction times (Ramogida and Orvig 2013; Price and Orvig 2014). The requirement to heat the current gold standard DOTA, above physiological temperatures for extended periods to facilitate incorporation of the ^225^Ac^III^ ion is adequate yet not ideal for small molecule or peptide bioconjugates that can withstand such temperatures but results in denaturing of sensitive biomolecules such as antibodies (Maguire et al. 2014; McDevitt et al. 2002). Consequently, two-step radiolabeling methods are often required for antibody radiolabeling. For example, the DOTA-isothiocyanate (DOTA-NCS) bifunctional chelate can be radiolabeled with ^225^Ac at elevated temperatures first, followed by conjugation to the antibody at mild temperature, unfortunately the hydrolysis of the isothiocyanate in the first step results in low radiochemical yields (8–11%) (McDevitt et al. 2002). Recently, Poty et al. have demonstrated that a DOTA-tetrazine bifunctional chelate can be radiolabeled in a two-step method to a transcyclooctene-modified antibody by exploiting the facile inverse electron-demand Diels-Alder reaction yielding bioconjugates with molar activities that are satisfying for TAT (Poty et al. 2018c). Nonetheless, a chelating ligand with the ability to quantitatively incorporate the ^225^Ac^III^ ion under conditions commensurate for radiopharmaceutical use (such as sub-micromolar ligand concentrations, mild ambient reaction temperatures, and fast reaction times), that also forms thermodynamically stable and kinetically inert complexes would be of high interest and utility in the field of ^225^Ac TAT. Furthermore, access to myriad chelate options will allow greater flexibility during future discovery work of ^225^Ac radiopharmaceuticals, since the radiometal-complex can be used to influence the pharmacokinetics of the overall construct, particularly for small molecule or peptide-based radiopharmaceuticals. In an effort to produce a viable alternative, an octadentate bispidine, H_2_bispa^2^ (Comba et al. 2017), and an 18-membered macrocycle, macropa (Thiele et al. 2017), have been reported that show improved properties for ^225^Ac radiolabeling, and to date are the only chelators that permit rapid, ambient temperature chelation (Deal et al. 1999; Chappell et al. 2000).

The aims, herein, were to (1) determine the feasibility of producing ^225^Ac via Isotope Separation On-Line (ISOL) at TRIUMF and (2) test the suitability of ISOL-derived ^225^Ac (in terms of its (radio) chemical properties and amount of radioisotope produced) to drive radiolabeling studies and preclinical radiopharmaceutical development. Within the second aim, we sought to answer two questions: (2a) can the ISOL-derived ^225^Ac be used to evaluate chelating ligands for Ac coordination; and (2b) is the ^225^Ac-ISOL product produced in sufficient amounts to drive preclinical radiopharmaceutical development? In terms of radiopharmaceutical development, a DOTA-bioconjugate was chosen since DOTA is currently the industry standard in ^225^Ac TAT.

**Fig. 1:**
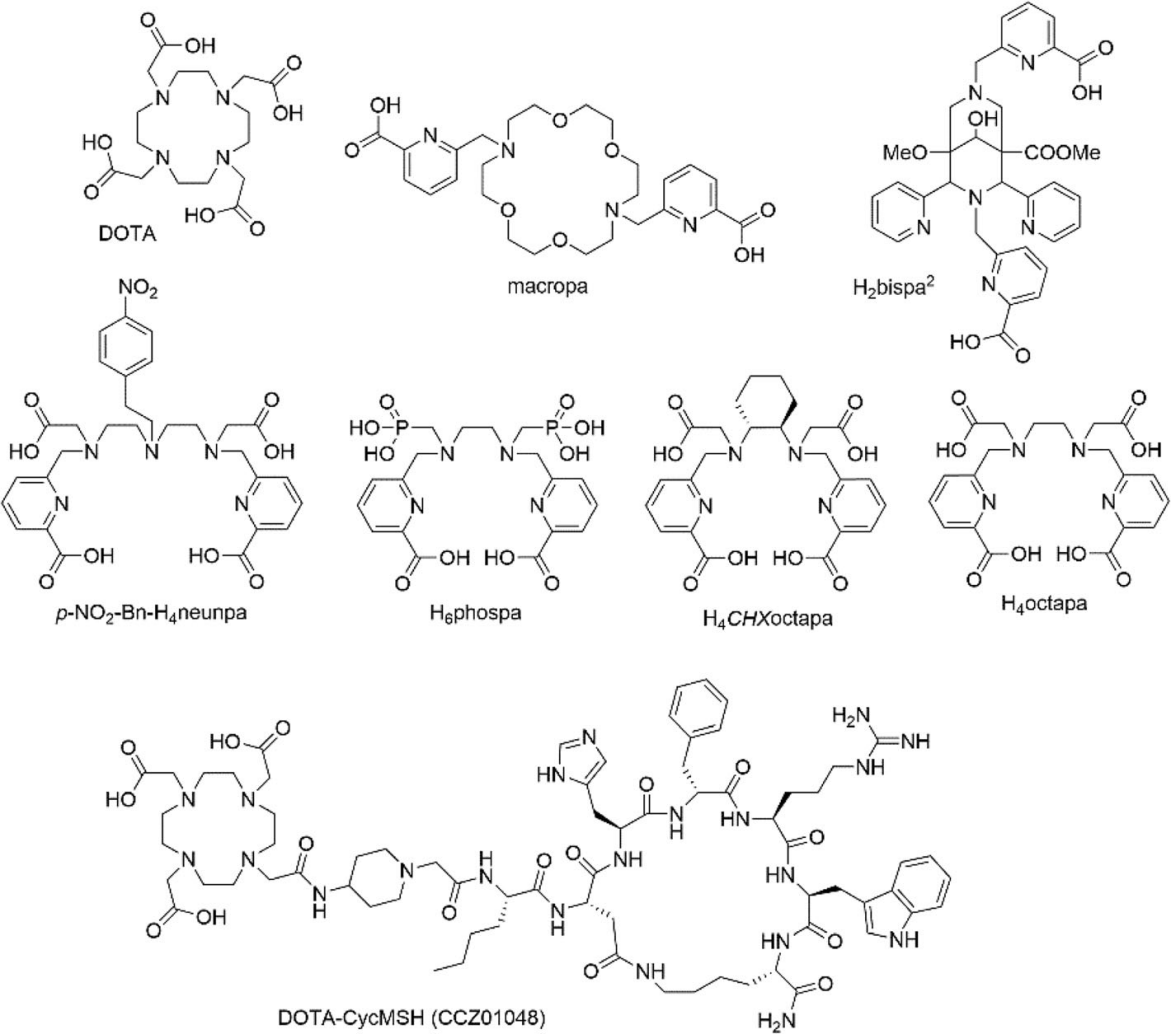
Ligand structures discussed in this work: macrocyclic commercial standard DOTA, previously reported ligands macropa and H_2_bispa^2^, acyclic picolinic acid “pa” ligands *p*-NO_2_-Bn-H_4_neunpa, H_6_phospa, H_4_*CHX*octapa, and H_4_octapa, and DOTA-CycMSH bioconjugate (CCZ01048)

ISOL facilities have been used to produce medical radionuclides that are otherwise challenging to obtain (Dilling et al. 2014; dos Santos Augusto et al. 2014; Crawford et al. 2017a; Crawford et al. 2017b; Crawford et al. 2018). Herein we report for the first time the ISOL-production of ^225^Ac, at ISAC, TRIUMF’s ISOL facility. The ^225^Ac isolated from these experiments was used in radiolabeling studies with a class of picolinic acid (“pa”) ligands developed in our laboratories (Price et al. 2014; Ramogida et al. 2015; Price et al. 2012; Spreckelmeyer et al. 2017) (Fig. 1) and compared to DOTA as well as macropa and H_2_bispa^2^ (Comba et al. 2017; Thiele et al. 2017). The “pa” chelating ligand family has demonstrated versatility for coordinating a number of imaging (e.g., gallium-67/68, indium-111, copper-64) and therapeutic isotopes (e.g., copper-64, lutetium-177, yttrium-90), making them extremely amenable for incorporation into a “theranostic” radiopharmaceutical. The octadentate and nonadentate “pa” ligands H_6_phospa (N_4_O_4_) (Price et al. 2014), *p-*NO_2_-Bn-H_4_neunpa (N_5_O_4_) (Spreckelmeyer et al. 2017), H_4_octapa (N_4_O_4_) (Price et al. 2012; Price et al. 2013), and H_4_*CHX*octapa (N_4_O_4_) (Ramogida et al. 2015) used in these radiolabeling experiments, were chosen because they were designed to incorporate larger metal ions such as actinium. Ac^III^ has a documented ionic radius of 112 ppm (coordination number, CN 6) (Shannon 1976) and is likely suited to large polydentate chelators of high denticities. In addition, two DOTA-peptide conjugates, DOTA-D-Phe^1^-Tyr^3^-octreotide (DOTA-TOC) (Graf et al. 2014; Eppard et al. 2017) and DOTA-Cyclized α-melanocyte-stimulating hormone (DOTA-CycMSH, Fig. 1) (Zhang et al. 2017) was radiolabeled with our ISAC derived ^225^Ac. Finally, a pilot in vivo study with the ^225^Ac-DOTA-CycMSH conjugate on tumour-bearing mice was performed to establish the feasibility of using ISOL-derived ^225^Ac to drive preclinical radiopharmaceutical development. Malignant melanoma is the most lethal form of skin cancer, accounting for 75% of deaths from this type of cancer. Receptor-targeted radiopharmaceuticals for melanoma imaging and therapy are promising strategies for diagnosis and treatment. In particular, the melanocortin-1 receptor (MC1R) is highly expressed in 83% of malignant melanoma cell lines, and has low expression in normal tissues making it an attractive target for radionuclide therapy and imaging (Zhang et al. 2017; Raposinho et al. 2010). Analogues of the tridecapeptide α-melanocyte-stimulating hormone (αMSH) have been studied extensively for MC1R-targeted imaging and less so for therapy. The most stable and successful analogues to date are based on lactam-bridged cyclized α-MSH (Ac-Nle4-cyclo [Asp^5^-His-D- Phe^7^-Arg-Trp-Lys^10^]-NH_2_, or Nle-CycMSH_hex_). Most recently our lab has developed a DOTA-CycMSH conjugate incorporating a cationic piperidine linker for positron emission tomography (PET) imaging with the positron-emitter gallium-68 (^68^Ga, t_1/2_ = 68 min) that exhibited promising ability to localize and internalize in melanoma cells, referred to herein as DOTA-CycMSH or CCZ01048 (Fig. 1). The [^68^Ga]Ga-CCZ01048 tracer exhibited high binding affinity to MC1R with sub-nanomolar K_i_ values, rapid internalization into B16F10 melanoma cells, high in vivo stability in blood plasma, and exceptional PET image contrast in vivo with tumour-to-blood, tumour-to-muscle, tumour-to-bone and tumour-to-kidney ratios of 96.4 ± 13.9, 210.9 ± 20.9, 39.6 ± 11.9 and 4.0 ± 0.9, respectively, at 2 h post-injection (Zhang et al. 2017). Given this ^68^Ga-tracer’s receptor binding affinity, rapid internalization, and low background organ uptake, we hypothesized that CCZ01048 would be a promising candidate for targeted alpha therapy with ^225^Ac, and biodistribution studies were therefore conducted to evaluate the ability of CCZ01048 to deliver ^225^Ac to B16F10 tumours in vivo.

## Materials and methods

All solvents and reagents were purchased from commercial suppliers (Fisher Scientific, Sigma Aldrich) and used as received. Ultrapure HCl (TraceSELECT®) and NaOH (99.99% trace metal grade) was purchased from Sigma Aldrich; nitric acid (TraceMetal™ Grade) was purchased from Fisher Scientific. Branched DGA resin (*N,N,N′,N′*-tetrakis-2-ethylhexyldiglycolamide, 50–100 μm particle size) was purchase from Eichrom Technologies (Lisle, IL, USA). Picolinate-based ligands H_4_octapa, H_4_*CHX*octapa, H_6_phospa, and *p*-NO_2_-Bn-H_4_neunpa were prepared as previously described (Price et al. 2014; Ramogida et al. 2015; Price et al. 2012), as were H_2_bispa^2^ and macropa (Comba et al. 2017; Thiele et al. 2017). DOTA and human serum were purchased from Sigma Aldrich (St. Louis, MO). Pharmaceutical grade DOTATOC was purchased from ABX, DOTA-CycMSH was prepared as previously described (Zhang et al. 2017). Deionized water (> 18 MΩ cm) was prepared on site using a Millipore Direct-Q® 3UV water purification system.

All radioactivity measurements were performed using gamma ray spectroscopy on an N-type co-axial high purity germanium (HPGe) gamma spectrometer (Canberra Industries) calibrated with a 20 mL ^152^Eu and ^133^Ba source. After thorough mixing, aliquots (5–100 μL) from a sample were removed and diluted to the 20 mL standard volume for measurement. Samples were counted at a distance at least 15 cm from the detector for at least 30 min or until peak areas were below 5%; dead time was kept below 5%. Spectra were analyzed using the Genie 2000 software package (version X, Canberra Industries), using 40 keV, 218 keV, and 440 keV gamma lines for ^225^Ra, ^221^Fr, and ^213^Bi, respectively. ^225^Ac was quantified either directly via its 188 keV emission, or indirectly via ^221^Fr after waiting at least 30 min to ensure equilibrium between ^225^Ac and ^221^Fr.

The High Performance Liquid Chromatography (HPLC) system used for analysis of ^225^Ac-bioconjugates consisted of an Agilent 1260 Infinity II Quaternary Pump, Agilent 1260 autosampler, Raytest Gabi Star NaI (Tl) radiation detector, Agilent 1260 variable wavelength detector, and Agilent 1260 analytical scale fraction collector. Phenomenex Luna 5 μm C18 100 Å liquid chromatography analytical (250 × 4.6 mm) and semi-preparative (250 × 10 mm) columns were used for the quality assurance analysis and purification of ^225^Ac-bioconjugates, respectively.

### ^225^Ac production and purification

Ion beams of ^225^Ra and ^225^Ac were created using the isotope separation on-line (ISOL) technique in operation at TRIUMF’s ISAC facility (Dilling et al. 2014). Detailed descriptions of the collection of radioactivity from these beams is described elsewhere (Robertson et al. 2018; Crawford et al. 2017a; Crawford et al. 2017b; Crawford et al. 2018; Kunz et al. 2018). Briefly, a uranium carbide target is bombarded with 9.8 μA of 480 MeV protons, which cause spallation, fission and fragmentation of the ^238^U target atoms. Simultaneous heating of the target under vacuum results in the diffusion and effusion of volatile reaction products out of the target into the transfer tube/ionizer region. The reaction products, among them ^225^Ra and ^225^Ac, are ionized via surface or resonant laser ionization and extracted as a secondary particle beam with an energy of 20–40 keV, which is subsequently mass separated and delivered to experiments – in our case an aluminum implantation target for isotope collection. The resolving power of the ISAC mass separator is such that the delivered A = 225 beam contains isotopes of Fr, Ra, and Ac. Fr, and to some degree Ac and Ra, ionize in the ISAC surface ion source; however, higher ionization efficiencies can be obtained by means of element selective resonant laser ionization. Efficient laser ionization schemes for Ac and Ra can now be applied at ISAC simultaneously for co-implantation of Ac and Ra. After implantation, the aluminum target containing the implanted radioactivity was removed from the beamline and the ^225^Ra and ^225^Ac retrieved from the aluminum material by etching the surface of the aluminum with 0.1 M HCl (~ 0.5 mL) as described previously (Kunz et al. 2018).

**Fig. 2:**
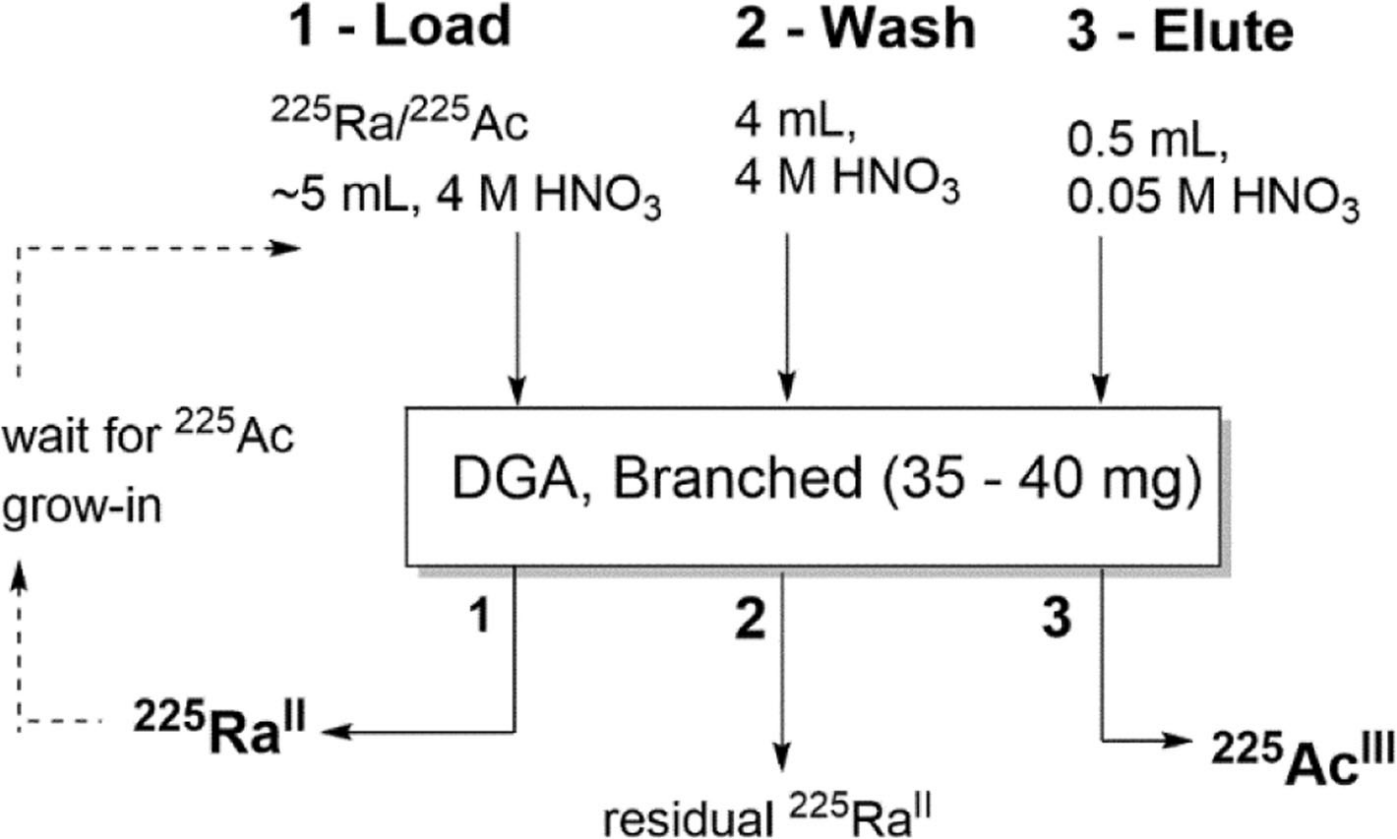
^225^Ra/^225^Ac radiochemical separation method used for purification of ISOL-produced ^225^Ac. The solution resulting from etching ^225^Ra and ^225^Ac from the aluminum target was diluted with nitric acid (to 4 M HNO_3_) and loaded onto a preconditioned DGA resin (Horwitz et al. 2005). After washing the resin (with 4 M HNO_3_) to remove residual ^225^Ra^II^, the ^225^Ac^III^ was eluted from the resin 0.5 mL of 0.05 M HNO_3_

The purification of ^225^Ac from ^225^Ra and other non-radioactive contaminants was adapted from previously reported methods for isolation of ^225^Ac from solid thorium targets (Zielinska et al. 2007; Apostolidis et al. 2005; Radchenko et al. 2015; Horwitz et al. 2005). Briefly, the recovered ^225^Ra/^225^Ac solution (0.1 M HCl, ~ 0.5 mL) was diluted to 4 M HNO_3_ (5 mL), and passed through a DGA column (35–40 mg of DGA branched resin in a 6 mm diameter reservoir, pre-conditioned with H_2_O (2 mL), 0.5 M HNO_3_ (2 mL), and 4 M HNO_3_ (2 mL)) (Horwitz et al. 2005). Under these conditions, Ac^III^ binds to the DGA while Ra passes through. The loaded DGA column was then washed with 4 M HNO_3_ (4 mL). After air drying the column, the ^225^Ac was eluted from the resin using 0.05 M HNO_3_ (0.5 mL). This separation scheme is illustrated in Fig. 2. The effluent (load fraction) containing ^225^Ra^II^ and the bulk of the non-radioactive Al^III^ impurities was collected and retained to decay, allowing for additional grow-in of ^225^Ac from ^225^Ra. Approximately every 1–3 weeks, the purification procedure was repeated to isolate a new batch of ^225^Ac.

The chemical purity of the ^225^Ac product was assessed via inductively coupled plasma optical emission spectrometry (ICP-OES) and inductively coupled plasma mass spectrometry (ICP-MS) to quantify the presence of any stable cations that may compete with ^225^Ac in radiolabeling reactions. Samples of both the initial solution containing ^225^Ra and ^225^Ac (before separation via DGA) and the final purified ^225^Ac solution were analyzed for elemental composition using an Agilent 7700× inductively-coupled plasma mass-spectrometer (ICP-MS). All samples were prepared by drying on a hotplate, treated with concentrated HNO_3_ and then again dried to convert all salts into the nitrate form. Samples were then diluted with a 1% HNO_3_, 0.05% HF solution containing 10 ppb Indium (internal standard). All acids used were sub-boiled to produce solutions containing a very low level of trace elements. Standard solutions were prepared from both mixed and single-element standards purchased from Inorganic Ventures (Christiansburg, VA, USA). Elements assayed included: Be, Al, Ca, Sc, Ti, Cr, Mn, Fe, Co, Ni, Cu, Zn, Ga, Sr, Y, Zr, Nb, Mo, Sn, W, and Pb.

### ^225^Ac radiolabeling studies and complex stability assays

The pro-ligands H_4_octapa, H_4_*CHX*octapa, H_6_phospa, *p*-NO_2_-Bn-H_4_neunpa and standard DOTA were made up as stock solutions (10 mg/mL, ~ 10^− 2^ M) in deionized water. A serial dilution was used to prepare ligand solutions at 10^− 3^, 10^− 4^, 10^− 5^, and 10^− 6^ M in water. A 10 μL aliquot of each ligand solution (or water, as a radiolabeling negative control) was diluted with ammonium acetate buffer (170 μL; 0.15 M; pH 5, 6, or 7). ^225^Ac(NO_3_)_3_ (5–30 kBq, 10–20 μL) was added and mixed gently to begin the radiolabeling reaction at ambient temperature (or 80 °C in the case of DOTA). Reaction pH (5.5 or 7) and temperatures (ambient or 40 °C) were applied and chosen because they are favourable conditions amenable for radiolabeling antibodies. An exhaustive list of radiolabeling reactions tested – repeated in triplicate – can be found in Additional file 1: Section S1.

With the exceptions of [^225^Ac(phospa)]^3−^ and [^225^Ac(neunpa)]^−^ complexes, for each stock ligand concentration and reaction pH, the reaction progress and radiochemical yield at varying time points (15–120 min) was analyzed by spotting a portion (1–5 μL) of the reaction mixture onto the bottom of an instant thin layer chromatography (iTLC) plate or aluminum backed silica TLC plate. After waiting at least 6 h to ensure equilibrium between ^225^Ac and its decay chain, plates were counted on a BioScan System 200 imaging scanner equipped with a BioScan Autochanger 1000.

Varying TLC/iTLC plate systems were selected for different ligands based from results of positive and negative radiolabeling controls:

- Method A, for [^225^Ac(octapa)]^−^ and [^225^Ac(*CHX*octapa)]^−^: iTLC-SG plates (Agilent, 2 cm × 10 cm, baseline at 1.5 cm), developed using 10 mM NaOH/9% NaCl solution. Under these conditions free ^225^Ac^III^ remains at the baseline (R_f_ = 0), while the ^225^Ac-ligand complex migrates upwards with the solvent (R_f_ > 0); or
- Method B, for [^225^Ac(bispa^2^)]^+^ and [^225^Ac(macropa)]^+^: aluminum-backed ultrapure silica gel 60 with 250 μm thickness TLC plates (Fisher, 2 cm × 10 cm, baseline at 1.5 cm), developed using 0.4 M sodium citrate (pH 4) with 10% methanol. Under these conditions free ^225^Ac^III^ migrates with the solvent front (R_f_ = 1), while ^225^Ac-ligand complex remains at the baseline (R_f_ = 0); (Comba et al. 2017; Thiele et al. 2017) or
- Method C, for [^225^Ac(DOTA)]^−^: iTLC-SA plates (Agilent, 2 × 10 cm, baseline at 1.5 cm) and developed using 50 mM EDTA buffer, pH 4. Under these conditions free ^225^Ac^III^ migrates with the solvent front (R_f_ = 1), while ^225^Ac-ligand complex remains at the baseline (R_f_ = 0); or
- Method D, for human serum stability (below): aluminum-backed ultrapure silica gel 60 with 250 um thickness TLC plates (Fisher, 2 cm × 10 cm, baseline at 1.5 cm), developed in 50 mM EDTA, pH 5. Under these conditions, chelate-bound ^225^Ac remains at the baseline (R_f_ = 0), and any ^225^Ac^III^ that has de-complexed from the chelate will travel with the solvent front (R_f_ = 1). Method D was validated by incubating “free” ^225^Ac^III^ with human serum under analogous conditions to ensure all the activity eluted with the solvent front (R_f_ ~ 1).

Radiolabeling yields of [^225^Ac(phospa)]^3−^ and [^225^Ac*(p*-NO_2_-Bn-neunpa)]^−^ were determined as follows: Briefly, to a 1 mL resin filled with Chelex (100–150 μg, sodium form, 50–100 mesh, equilibrated with NH_4_OAc [0.15 M, pH 5.5]), the entire radiolabeling reaction mixture was loaded (Full). The resin was eluted with NH_4_OAc (0.15 M, pH 5.5, 2 mL) (Eluted) to collect the ^225^Ac-complex while “free” unbound ^225^Ac^III^ remained on the resin. The eluate was diluted to 20 mL with H_2_O in a scintillation vial and activity quantified via HPGe gamma spectroscopy. The radiolabeling yield was calculated by comparing the fraction of ^225^Ac collected in the eluate (Elute) with the amount in the initial radiolabeling reaction (Full); i.e. % RCY = (Elute)/(Full)*100.

To investigate the stabilities of the ^225^Ac-complexes, a competition experiment was performed in the presence of excess human serum and the displacement of radioactivity from the chelator to serum proteins was tracked over the course of seven days. Endogenous metal-binding proteins in serum such as *apo*-transferrin and metallothionein can compete for and displace chelator-bound metal ions in vivo, preventing successful delivery of the radionuclide to the desired target. The ^225^Ac-complexes were prepared using the radiolabeling protocols described above and added to an equal volume (170–180 μL) of human serum (stored at − 5 °C and thawed at ambient temperature). At varying time points (0.5 h to 7 days), small aliquots (1–5 μL) were spotted on iTLC plates using Methods B or D, described above. Representative TLC radio-chromatograms of a serum competition assay with ligand and control are presented in Additional file 1: Figures S2 and S3.

The stability of a radiometal-chelate complex is also often assessed by its ability to withstand transchelation in the presence of excess stable isotopes of the radiometal. Though this is an impossible experiment to perform with Ac since all Ac isotopes are unstable, lanthanum(III) has a similar ionic radius (103–116 ppm, CN = 6–9) and charge to actinium(III) and is often considered a chemical surrogate to Ac^III^. Using non-radioactive metal ions such as La^III^ as a competitor for ligand complexation is a straightforward method to probe the kinetic off-rate of a pre-formed Ac^III^ complex, since many ligands that bind Ac^III^ may also efficiently complex La^III^. Herein, ^225^Ac-complexes were prepared using the radiolabeling protocols described above and combined with La(NO_3_)_3_ (0.1 M, 50 M equivalents compared to the ligand) and allowed to react at ambient temperature. At varying time points (1 h – 7 d), aliquots of the competition reaction mixture (1–5 μL) were spotted on aluminum backed TLC plates and developed using the TLC methods described above.

### Bioconjugate labeling studies and pilot in vivo studies with ^225^Ac-DOTA-CycMSH

Preliminary radiolabeling studies with DOTATOC using ISOL-derived ^225^Ac was performed. To prepare ^225^Ac-labeled DOTATOC, ^225^Ac^III^ (37 kBq or 1.5 MBq) was added to a vial containing precursor (25 μg, in 25 μL deionized water) and ammonium acetate buffer (1 M, pH 7) to give a final ligand concentration between 19 and 9.8 × 10^− 5^ M and heated at 85 °C for 40 min. Reaction progress and RCYs were determined using iTLC method B (see ^225^Ac radiolabeling studies and complex stability assays section).

To further demonstrate the utility of ISAC-ISOL derived ^225^Ac and to explore the potential of novel ^225^Ac-radiopharmaceuticals, preliminary radiolabeling and in vivo studies were conducted with a previously reported lactam bridge-cyclized α-melanocyte-stimulating hormone peptide conjugated to the DOTA chelator (DOTA-CycMSH or CCZ01048) (Fig. 1).

To prepare ^225^Ac-labeled CCZ01048, the entire ^225^Ac fraction from a ^225^Ra/^225^Ac separation (see ^225^Ac production and purification section) was evaporated to dryness and reconstituted with 25 μL of 0.05 M HNO_3_. This ^225^Ac solution was combined with CCZ01048 (20–80 μg, 25 μL in deionized water) and an ammonium acetate buffer (1 M, pH 7, 35 μL). The reaction mixture was subsequently heated to 85 °C for 30 min, and reaction progress and RCYs determined using iTLC Method B (see ^225^Ac radiolabeling studies and complex stability assays section). More details regarding the selection of conditions for labeling CCZ01048 with ISAC-derived ^225^Ac can be found in Additional file 1: Section S2.

Following the radiolabeling reaction for the non-blocked in vivo experiments, HPLC purification was performed to remove unbound ^225^Ac^III^ and to separate radiolabeled product from precursor to ensure high molar activity of the final product for preclinical biodistribution studies. The reaction mixture was separated by HPLC using a semi-preparative column eluted with 22% acetonitrile containing 0.1% TFA for ^225^Ac-CCZ01048 at a flow rate of 4.5 mL/min. The retention time of the ^225^Ac-labeled peptide was 9.5 min, compared to 10.0 min for the unlabeled peptide. The collected radiolabeled peptide was diluted with deionized water and retained on a C18 SepPak cartridge (Waters, preconditioned with 5 mL ethanol and 10 mL deionized water) to remove acetonitrile and TFA. The purified peptide was eluted with ethanol and diluted with 0.9% NaCl saline. HPLC purification was not performed for the blocking studies, but instead the radiolabeling reaction was diluted with ammonium formate (0.05 M, 10 mL), loaded onto a pre-conditioned C18 SepPak to remove unlabelled ^225^Ac^III^, and the purified peptide was eluted with ethanol and diluted with 0.9% NaCl. Quality control of both reactions was conducted by iTLC-SG developed in citric acid (0.05 M, pH 5); under these conditions unbound ^2225^Ac^III^ travels with the solvent front (R_f_ = 1) while ^225^Ac-bound to peptide remains at the baseline (R_f_ = 0). Both ^225^Ac tracers had radiochemical purity > 98%. To determine the final molar activities of the purified tracer prior to in vivo injection, an aliquot of known volume (5–10 μL) was counted on the HPGe gamma spectrometer, and ^225^Ac activity per unit volume was calculated. In parallel, a known volume of final diluted tracer was analyzed using analytical HPLC eluted with 22% acetonitrile containing 0.1% TFA at a flow rate of 1 mL/min, and the amount of peptide was quantified by integrating the peaks in the UV-Vis spectra (at 220 nm) and comparing to a standard curve of the [La(CCZ01048)] complex. The retention times of the unlabeled peptide and La-complex were 11.4 and 10.8 min, respectively.

The B16F10 melanoma cell line (*Mus musculus*) used in the tumour model was purchased from ATTC (CRL-6475), and handled as previously described (Zhang et al. 2017). The cell line was confirmed pathogen-free by the IMPACT 1 mouse profile test (IDEXX BioResearch). Cells were cultured in Dulbecco’s Modified Eagle’s Medium (DMEM, StemCell Technologies) supplemented by 10% FBS, 100 U/mL penicillin and 100 μg/mL streptomycin at 37 °C in a humidified incubator containing 5% CO_2_. Cells grown to approximately 90% confluence were washed with sterile 1× PBS (pH 7.4), followed by trypsinization.

Biodistribution studies were performed using male C57BL/6 J mice. For tumour implantation, mice were anesthetized using isoflurane on a precision vaporizer (5% for induction, and 2% for maintenance) in 2.0 L/min of oxygen, and 1 × 10^6^ B16F10 cells were implanted subcutaneously on the right back at the level of the forelimbs. Tumours were allowed to grow and reach approximately 9 mm in diameter and used for biodistribution studies. Mice were anesthetized, and injected with 12–20 kBq of ^225^Ac-CCZ01048 prepared at either high molar activity (> 200 kBq/nmol for non-blocking, *n* = 4) or low molar activity (1.2 kBq/nmol for blocking studies, *n* = 4).

After injection, the mice were allowed to roam freely in their cages and sacrificed 2 h post-injection by *CO*_*2*_ asphyxiation under isoflurane *anesthesia*. Cardiac puncture was promptly performed to recover blood, and the organs of interest were harvested, rinsed with 1× PBS, and blotted dry. Each organ was weighed, and the radioactivity of the collected tissue was measured using a calibrated gamma counter 931(Packard Cobra II Auto-gamma counter, Perkin Elmer, Waltham, MA, USA) using three energy windows: 60–120 keV (window A), 180–260 keV (window B), and 400–480 keV (window C). Counting was performed both immediately following sacrifice and after 7 days to ensure equilibrium of the ^225^Ac decay chain (Note: secular equilibrium of ^225^Ac with its daughters is reached at about 24 h; samples in this study were counted after 7 days due to availability of the gamma counter). Counts were decay corrected from the time of injection and total organ weights were used for the calculation of injected dose per gram of tissue (%ID/g). No differences were noted between the data; therefore, the biodistributions are reported using the data acquired using window A.

## Results

### ^225^Ac production and purification

Mass A = 225, singly-charged (+ 1) ion beams produced at TRIUMF’s ISAC facility contained ^225^Ra and ^225^Ac intensities between 4.0 × 10^6^–1.6 × 10^8^ and 3.8 × 10^6^–1.3 × 10^8^ ions/s, respectively, depending on the state of the extraction electrode and the availability of the laser ionisation source. ^225^Fr (t_1/2_ = 4.0 min) was also present but decayed rapidly to ^225^Ra. For durations between 13.3–80.7 h, the 40 keV ions beams were implanted into an aluminum disk to a depth of 15–25 nm as determined by SRIM (Ziegler et al. 2010). Etching of the radioactivity from the aluminum target post-implantation with 500 μL of 0.1 M HCl resulted in 0.2–7.5 MBq of ^225^Ra and 0.16–18.0 MBq of ^225^Ac. A summary of the production runs is shown in Table 1.

**Table 1:** Summary of ^225^Ra and ^225^Ac production runs at ISAC, TRIUMF’s ISOL facility. ^a^EE = extraction electrode; ^b^LIS = laser ionization source

Implantation parameters				Ion beam intensity [ions/s]		Activity Produced [MBq]	
Date	duration [h]	EE^*a*^	LIS^*b*^	^225^Ra	^225^Ac	^225^Ra	^225^Ac
Dec ‘15	13.3	Off	Off	3.2 × 10^7^	3.8 × 10^6^	0.19	0.16
Apr ‘16	44.8	Off	On	4.0 × 10^6^	1.0 × 10^7^	0.99	1.40
May ‘16	48.9					1.13	1.35
Aug ‘16	21.6	On	On	1.6 × 10^8^	5.7 × 10^7^	7.1	10.5
Dec ‘16	45.0	On	On	9.3 × 10^7^	1.3 × 10^8^	6.8	18.0
Apr ‘17	80.7	Off	On	9.0 × 10^7^	2.8 × 10^6^	7.5	1.7
Sept ‘18	~ 40	On	Off	1.0 × 10^8^	1.6 × 10^7^	8.6	9.4

**Fig. 3:**
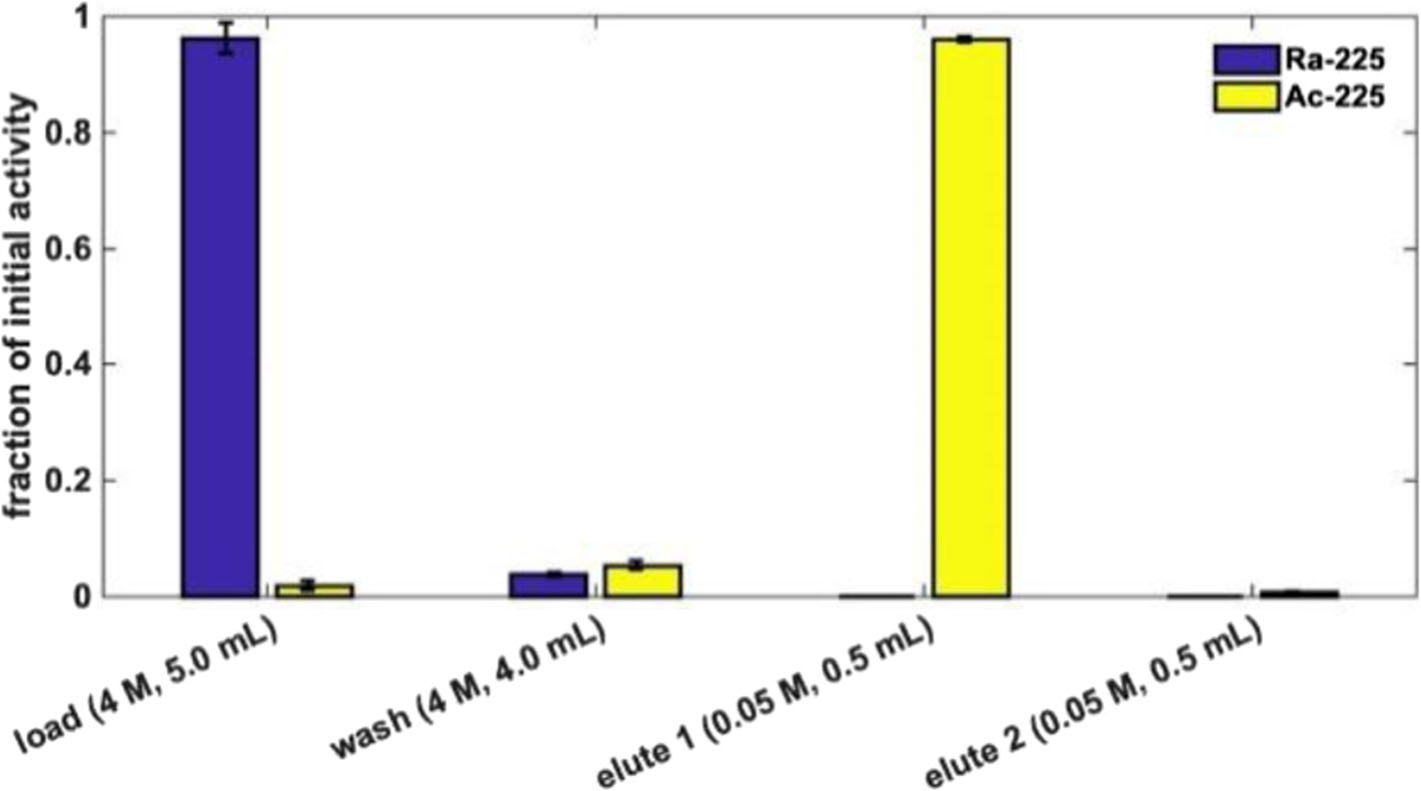
Representative elution profile for ^225^Ra/^225^Ac separation on DGA branched resin in nitric acid media

One-step purification of ^225^Ac from ^225^Ra on the DGA column reliably yielded a pure ^225^Ac product, free from parent isotope ^225^Ra, with yields > 90% as determined by gamma ray spectroscopy. A second elution with an additional 0.5 mL of 0.05 M HNO_3_ is typically able to recover the remaining ^225^Ac. The overall purification yield of ^225^Ac from the target solution was > 98%. Example results from a single separation are shown in Fig. 3. The load fraction containing ^225^Ra was retained and used as an ^225^Ac generator, providing a supply of ^225^Ac for nearly 3 months.

To quantify the amount of stable metal impurities present in the ^225^Ac product that could compete with ^225^Ac during radiolabeling reactions, the chemical purity of the ^225^Ac was assessed via ICP-OES and ICP-MS. Modest values of aluminum with 7709 ± 1281 and 10175 ± 872 ppb (3.9 ± 0.6 and 5.1 ± 0.4 μg) were present in the first and second eluates, respectively, compared to 166,000 ppb (830 μg) present in the load fraction (Table 2). The ~ 200-fold decrease of Al in the eluate 1 compared to the initial load solution suggests that the DGA resin does a satisfactory job of removing excess Al^III^ from the ^225^Ac^III^ product with a separation factor of ~ 10^2^; however, an additional washing step may further decrease the amount of metal impurity, which may in turn positively impact radiolabeling yields. Calcium, iron, copper, nickel and zinc were found in the ^225^Ac product (elute 1) with amounts of 1392 ± 208, 203 ± 20, 47 ± 12, 19 ± 6, and 137 ± 21 ppb, respectively. If one assumes the entire solution of eluate 1 (0.5 mL) were to contain 10 MBq of ^225^Ac, the non-radioactive impurities would account for 6970 ± 1158 atoms of aluminum, 847 ± 127 atoms of calcium, 88 ± 9 atoms of iron, 51 ± 8 atoms of zinc, 8 ± 2 atoms of nickel, and 18 ± 5 atoms of copper per atom of actinium. Of concern are the high M^n+^:Ac^III^ ratios of Al^III^, Fe^III^, and Ca^II^ in the ^225^Ac eluate, since the gold standard in actinium chelation, DOTA, also has a strong affinity for these metals (Clarke and Martell 1991). Additional ICP-MS results for other elements (in ppb and μg) can be found in Additional file 1: Section S3.

**Table 2:** Trace metal content in ppb (μg/L) determined by ICP-MS (*n* = 2)

Fraction	Al	Ca	Fe	Cu	Ni	Zn
Load	166000^a^	47.4 ± 0.8	274 ± 45	32 ± 11	18 ± 15	57 ± 25
Wash	14000^a^	63 ± 8	94 ± 3	12 ± 5	76 ± 94	36 ± 5
Elute 1	7709 ± 1281	1392 ± 208	203 ± 20	47 ± 12	19 ± 6	137 ± 21
Elute 2	10175 ± 872	745 ± 24	427 ± 192	46 ± 5	37 ± 17	134 ± 23

^a^ values determined by ICP-OES, *n* = 1

### ^225^Ac radiolabeling studies

The phosphinic acid ligand (phospa)^6−^ was able to efficiently complex ^225^Ac^III^ (radiochemical yield, RCY 95%) in one hour at ambient temperature, pH 5.5, and 10^− 3^ M ligand concentrations; with decreasing ligand concentrations (10^− 4^, 10^− 5^, 10^− 6^ M), RCYs dropped sequentially from 82, 14, to 0%, respectively. At even 10^− 3^ M ligand concentration, nonadentate ligand (*p*-NO_2_-Bn-neunpa)^4−^ displayed only a moderate radiochemical yield (56%) after 1 h at ambient temperature. Heating increased the RCY to 87% under the same ligand concentration and time (Additional file 1: Table S1).

The octadentate ligand (octapa)^4−^ efficiently complexed (RCY > 95%) ^225^Ac^III^ at 10^− 4^ and 10^− 5^ M ligand concentrations after one hour at ambient temperature, while the RCY dropped to 7% at a ligand concentration of 10^− 6^ M. After only 30 min, radiolabeling was determined to be incomplete even at the 10^− 4^ M ligand concentration (RCY = 77%), and thus 60-min timepoints were used to test the remaining labeling reactions for (octapa)^4−^. The chiral derivative, (*CHX*octapa)^4−^ was able to efficiently complex ^225^Ac^III^ at ambient temperature in one hour at 10^− 4^, 10^− 5^ and 10^− 6^ M, exhibiting RCYs of 91, 94 and 94%, respectively; at ligand concentration of 10^− 7^ M, RCY decreased to 4%. Radiochemical yields > 90% were also obtained at 10^–4 to − 6^ M ligand after 30 min.

DOTA was inefficient at complexing ^225^Ac^III^ at ambient temperature, and required heating to 85 °C to induce ^225^Ac^III^ labeling (Additional file 1: Table S1).

**Fig. 4:**
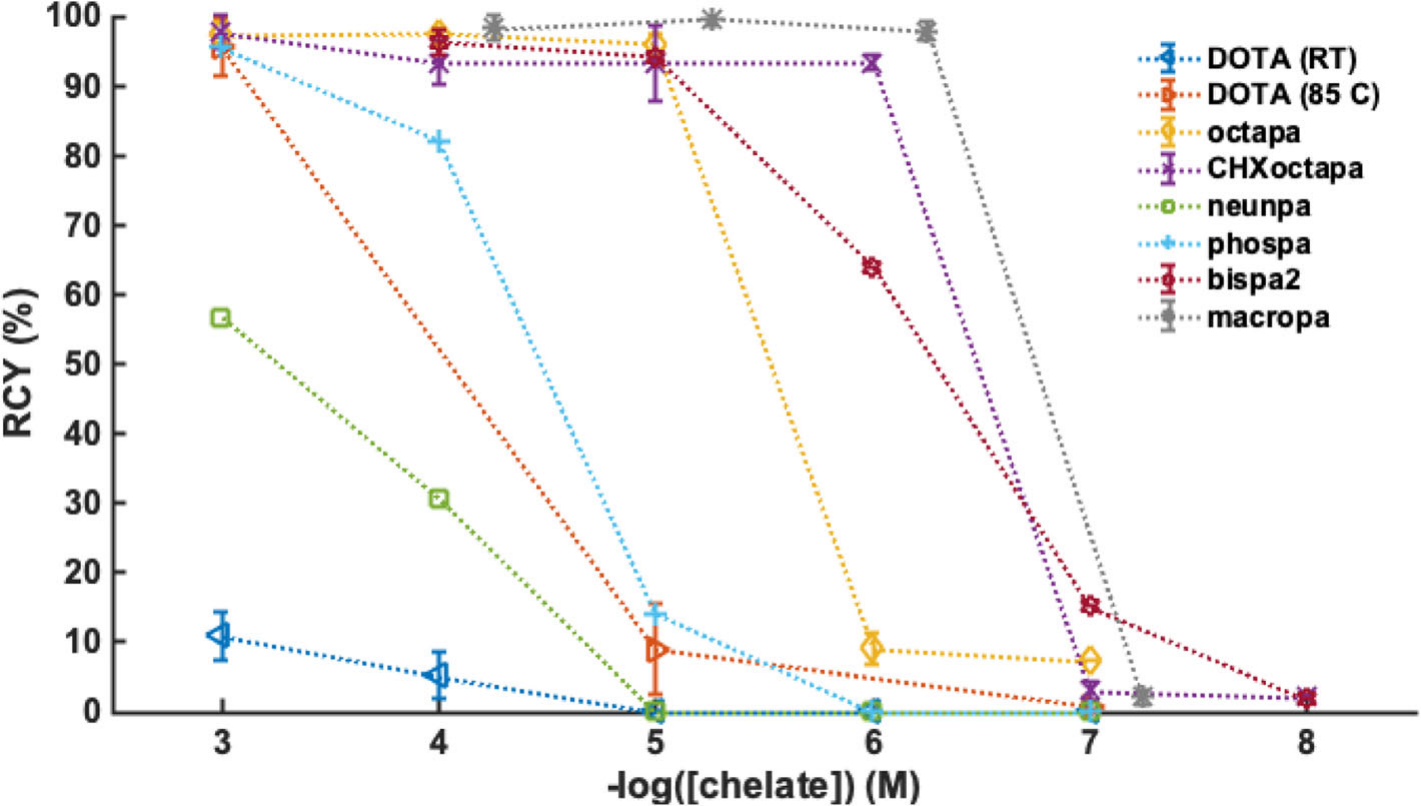
Radiochemical yields (RCY, %) for ^225^Ac^III^ radiolabeling reactions of DOTA (RT, 2 h, pH 5.5; 85 °C, 30 min, pH 7, H_4_octapa (RT, 1 h, pH 5.5), H_3_*CHX*octapa (RT, 1 h, pH 5.5), H_4_neunpa (RT, 1 h, pH 5.5), H_6_phospa (RT, 1 h, pH 5.5), H_2_bispa^2^ (RT, 1 h, pH 5.5) and macropa (RT, 5 min, pH 7) for comparison, at ambient temperature (RT) and ligand concentrations 10^− 3^ to 10^− 8^ M

Previously published ^225^Ac radiolabeling results with ligands (macropa)^2−^ and (bispa^2^)^2−^, (Comba et al. 2017; Thiele et al. 2017), which were performed with the same source of ^225^Ac are also plotted in Fig. 4 for comparison. As previously reported, octadentate bispidine, (bispa^2^)^2−^, was able to efficiently complex ^225^Ac after one hour at ambient temperature with ligand concentrations of 10^− 4^ and 10^− 5^ M (RCYs of 98 and 94%, respectively). At ligand concentrations of 10^− 6^, 10^− 7^, and 10^− 8^ M, ^225^Ac RCYs decreased sequentially to 64, 15, and 2%, respectively. Unlike the gold standard DOTA and the rest of the tested pro-ligands (H_4_octapa, H_4_*CHX*octapa, *p*-NO_2_-Bn-H_4_neunpa, H_6_phospa), the 18-membered macrocycle, macropa, can quantitatively complex ^225^Ac (RCYs > 95%) at ligand concentrations as low as 10^− 7^ M, in 5 min at ambient temperature (Thiele et al. 2017).

The stability of ^225^Ac complexes in human serum for [^225^Ac(octapa)]^−^, [^225^Ac(*CHX*octapa)]^−^, [^225^Ac(phospa)]^3−^ [^225^Ac(DOTA)]^−^, [^225^Ac(bispa^2^)]^+^, and [^225^Ac(macropa)]^+^ is shown in Table 3. The inability of *p-*NO_2_-Bn*-*H_4_neunpa to quantitatively complex ^225^Ac^III^ under any tested conditions precluded us from testing the stability of the resulting [^225^Ac(*p-*NO_2_-Bn*-*neunpa)]^−^ complex. The [^225^Ac(octapa)]^−^ and [^225^Ac(*CHX*octapa)]^−^ complexes exhibited favourable stabilities in human serum, remaining 92.9 ± 1.0 and 95.6 ± 1.6% intact after 7 days, respectively, while [^225^Ac(phospa)]^3−^ was moderately stable in serum, remaining 77.1 ± 6.4% intact after 7 days. The commercial standard [^225^Ac(DOTA)]^−^ remained sufficiently intact after 7 days (84.7 ± 8.1%). Both the previously reported [^225^Ac(bispa^2^)]^+^ and [^225^Ac(macropa)]^+^ complexes, which remained 88.9 ± 2.8 and 90% intact after 7 days in serum, respectively, show promising complex stabilities (Thiele et al. 2017), but slightly lower than the [^225^Ac(octapa)]^−^ and [^225^Ac(*CHX*octapa)]^−^ complexes.

**Table 3:** Stability of ^225^Ac-labelled chelate complexes in human serum at ambient Temperatures (*n* = 3 unless otherwise noted)

^225^Ac-complex	Time Point							
% Stable	0.5 h	1 h	4 h	1 d	2 d	3 d	4 d	7 d
[^225^Ac(octapa)]^−^	ND^a^	97.9 ± 0.9	96.9 ± 1.5	94.9 ± 1.2	96.1 ± 1.0	94.9 ± 1.1	95.9 ± 1.9	92.9 ± 1.0
[^225^Ac(*CHX*octapa)]^− b^	ND^a^	95.9 ± 1.0	96.8 ± 1.7	97.0 ± 0.9	96.7 ± 1.6	96.1 ± 1.5	96.7 ± 1.2	95.6 ± 1.6
[^225^Ac(phospa)]^3−^	ND^a^	81.6 ± 1.1	ND^*a*^	81.5 ± 0.2	ND^*a*^	80.2 ± 1.1	76.2 ± 0.1	77.1 ± 6.4
[^225^Ac(DOTA)]^−^	92.4 ± 4.0	93.9 ± 4.5	94.4 ± 3.7	90.9 ± 5.6	91.8 ± 5.6	ND^*a*^	91.1 ± 6.1	84.7 ± 8.1
[^225^Ac(macropa)]^+^ (Thiele et al. 2017) ^c^	96	95	93	90	93	90	ND^*a*^	90
[^225^Ac(bispa^2^)]^+^ (Comba et al. 2017)	ND^a^	97.4 ± 0.5	ND^*a*^	92.3 ± 0.7	ND^*a*^	90.7 ± 0.5	ND^*a*^	88.9 ± 2.8
Control	1.45 ± 3.8	1.29 ± 4.0	1.6 ± 3.5	2.6 ± 5.0	3.9 ± 3.0	ND^*a*^	0.96 ± 3.3	1.5 ± 4.1

^a^*ND* not determined; ^b^*n* = 4; ^c^*n* = 1

The results of the La^III^ exchange competition assay are summarized in Table 4 and compared to previously reported values for [^225^Ac(macropa)]^+^ and [^225^Ac(bispa^2^)]^+^ (Comba et al. 2017; Thiele et al. 2017). The ^225^Ac^III^ was readily displaced from the [^225^Ac(phospa)]^3−^ complex upon addition of excess La^III^. After 1 h, none of the ^225^Ac^III^ remained complexed to (phospa)^6−^, suggesting that (phospa)^6−^ does not form kinetically inert complexes with Ac^III^ and readily forms complexes with La^III^. Significant decomplexation of the [^225^Ac(octapa)]^−^ complex was observed over a short period of time, with 78.9 ± 2.9, 42.7 ± 0.3, 4.7 ± 1.5, 6.1 ± 2.6% of the complex remaining intact after 1 h, 5 h, 1 d, and 2 d, respectively. The [^225^Ac(*CHX*octapa)]^−^ complex displayed slightly delayed decomplexation kinetics compared to the (octapa)^4−^ complex, with 81.8 ± 4.3, 45.7 ± 3.1, 26.5 ± 8.9, 18.1 ± 0.4, 5.0 ± 0.4, 3.9 ± 0.6% intact ^225^Ac-complex remaining after 5 h, 1, 2, 3, 6, and 7 d, respectively. The cyclohexyl backbone of H_4_*CHX*octapa was designed to pre-organize the donor atoms of the linear chelating ligand, in hopes to form more kinetically inert complexes compared to the achiral analogue H_4_octapa (Ramogida et al. 2015). The results of the La^III^ exchange study suggest that the introduction of the cyclohexyl diamine backbone indeed delay the kinetics of exchange of (*CHX*octapa)^4−^ and its metal complexes compared to (octapa)^4−^ complexes, though neither [^225^Ac(octapa)]^−^ or [^225^Ac(*CHX*octapa)]^−^ were able to sufficiently withstand transchelation at later time points. In contrast, [^225^Ac(macropa)]^−^, [^225^Ac(DOTA)]^−^, and [^225^Ac(bispa^2^)]^−^ remained 91, 77.1 ± 3.6, and 71.1 ± 2.7% intact after 7 days. In summary, taking into consideration the results from the La^III^ exchange competition assay, a trend in the robustness of the resulting ^225^Ac-complexes can be drawn as follows: (phospa)^6−^ < (octapa)^4−^ < (*CHX*octapa)^4−^ < (bispa^2^)^2−^ ~ (DOTA)^4−^ < (macropa)^2−^.

**Table 4:** Stability of ^225^Ac-Labelled Chelate Complexes in 50-fold excess La^III^ at ambient temperature

Ligand	% stable						
Time point:	1 h	5 h	1 d	2 d	3 d	6 d	7 d
[^225^Ac(octapa)]^−^ (*n* = 2)	78.9 ± 2.9	42.7 ± 0.3	4.7 ± 1.5	6.1 ± 2.6	ND^a^	ND^a^	ND^a^
[^225^Ac(CHXoctapa)]^−^ (*n* = 2)	ND^a^	81.8 ± 4.3	45.7 ± 3.1	26.5 ± 8.9	18.1 ± 0.4	5.0 ± 0.4	3.9 ± 0.6
[^225^Ac(phospa)]^3−^ (*n* = 3)	0	0	ND^a^	ND^a^	ND^a^	ND^a^	ND^a^
[^225^Ac(DOTA)]^−^ (*n* = 2)	95.2 ± 3.3	94.5 ± 3.5	94.9 ± 2.9	86.2 ± 3.1	ND^a^	ND^a^	77.1 ± 3.6
[^225^Ac(macropa)]^+^ (Thiele et al. 2017)	95	ND^a^	93	92	91	93	91^b^
[^225^Ac(bispa^2^)]^+^ (Comba et al. 2017)	ND^a^	95.5 ± 0.2	91.5 ± 0.7	88.8 ± 2.5	85.2 ± 1.7	74.4 ± 1.4	71.1 ± 2.7

^a^*ND* not determined. ^b^8 d time point

### Bioconjugate labeling studies and pilot in vivo studies with ^225^Ac-DOTA-CycMSH

The DOTA-bioconjugates, DOTATOC were radiolabeled with ISOL-derived ^225^Ac with moderate to poor yields. After heating at 85 °C for 40 min, [^225^Ac]Ac-DOTATOC radiolabeling yields of 91 ± 5% (*n* = 3, 37 kBq ^225^Ac used per reaction) or 43 ± 14% (*n* = 3, 1.5 MBq ^225^Ac used per reaction) were obtained, heating for longer periods of time did not increase radiolabeling yield.

[^225^Ac]Ac-CCZ01048 radiotracers were prepared with molar activities of > 200 kBq/nmol or 1.6 kBq/nmol, for non-blocked and blocked in vivo biodistribution experiments, respectively. Results from CCZ01048 radiolabeling studies with varying concentrations of ^225^Ac and precursor are shown in Additional file 1: Section S2. In general, high ^225^Ac radiolabeling yields (> 95%) of CCZ01048 were obtained when 60 μg (39 nmol) or more precursor was added, and radiolabeling yields dropped dramatically (57%) when 50 μg (33 nmol) of precursor was used.

**Fig. 5:**
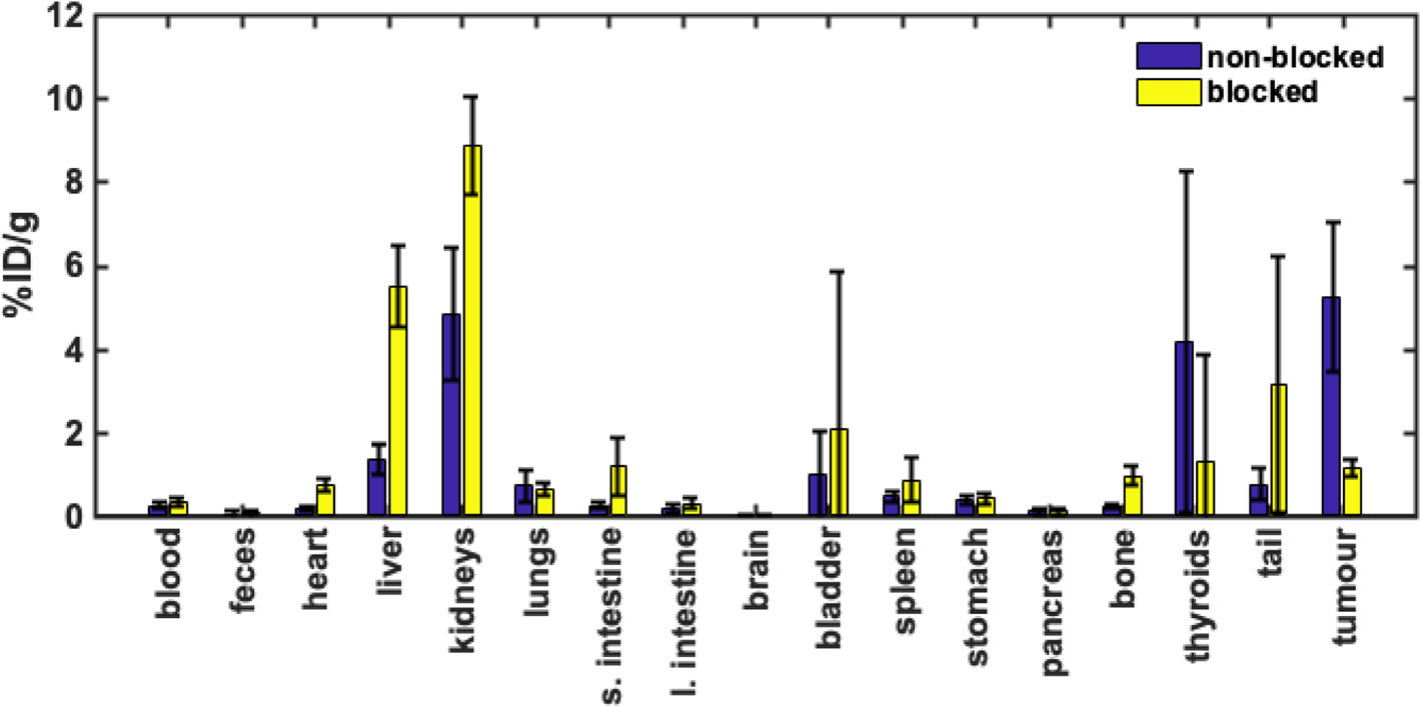
Blocked and non-blocked biodistribution results (both *n* = 4) of [^225^Ac]Ac-CCZ01048 at 2 h post-injection

Biodistribution results of the non-blocked experiment revealed tumour uptake at 2 h post injection of 5.23 ± 1.78%ID/g, with tumour-to-blood, tumour-to-bone, and tumour-to-kidney ratios of 20.42 ± 3.38, 23.16 ± 10.32, and 1.09 ± 0.11%ID/g, respectively (see Table 5). Uptake in other organs is shown in Fig. 5. To confirm the tumour uptake was receptor mediated, a blocking experiment was conducted by injecting low molar activity ^225^Ac-tracer (1.6 kBq/nmol) in tumour-bearing mice. As expected tumour uptake at 2 h post-injection decreased to 1.15 ± 0.21%ID/g, while kidney uptake increased to 8.85 ± 1.19% ID/g (compared to 4.83 ± 1.58%ID/g from non-blocked experiment). These results agree with the previously reported ^68^Ga-labeled tracer (Zhang et al. 2017).

**Table 5:** Summary of in vivo biodistribution studies of [^225^Ac]Ac-CCZ01048 at 2 h post-injection. Statistical analysis between non-blocked and blocked experiments was performed using the Student’s *t*-test (* *p <* 0.05; ** *p* < 0.005; *n* = 4 for each animal experiment)

Study	Molar activity (kBq/nmol)	Tumour (%ID/g)	Kidney (%ID/g)	Blood (%ID/g)	Tumour: blood	Tumour: bone	Tumour: kidney
Non-blocked	> 200	5.23 ± 1.78	4.83 ± 1.58	0.25 ± 0.07	20.42 ± 3.38	23.16 ± 10.32	1.09 ± 0.11
Blocked	1.6	1.15 ± 0.21**	8.85 ± 1.19*	0.34 ± 0.09	3.50 ± 0.72**	1.22 ± 0.27**	0.13 ± 0.04**

## Discussion

^225^Ac obtained from TRIUMF’s ISOL facility was purified using a simple method with a high yield (> 98%), high radionuclidic purity (> 98%) and chemical purity suitable for preclinical screening of potential targeted alpha therapy compounds (Comba et al. 2017; Thiele et al. 2017). The entire purification process, from target dissolution to radiochemical purification is typically completed in under 2 h and produces less than 10 mL of radioactive liquid waste. In comparison, the current supply of ^225^Ac which is separated from a ^229^Th stock requires at least four separate column purifications to remove the multi-gram quantities of Th from Ra/Ac, evaporation procedures, and produces several litres of radioactive liquid waste with anywhere between 85 and 95% Ac recovery yields and > 99% radionuclidic purity (Robertson et al. 2018). For a detailed discussion of the current production and purification methods of ^225^Ac the reader is referred to the following review article (Robertson et al. 2018). Since the mass separator is of high resolution and efficiency, ISOL derived ^225^Ac can provide a radiochemically pure product without the need to chemically separate from multi-gram Th quantities or other target materials, allowing facile and rapid ^225^Ac purification using minimal solvent volumes. The main current disadvantage of the ISOL method for ^225^Ra/^225^Ac production is the limited yield that can be produced; the highest amount of ^225^Ac and ^225^Ra was 18.0 MBq and 8.6 MBq, respectively, from any one implantation (not including additional ^225^Ac activity isolated from the ^225^Ra generator).

Potential improvements to this ISOL ^225^Ac source are possible and include: using higher proton beam currents (up to 100 μA) to increase ^225^Ra and ^225^Ac production inside the uranium target; increasing ISAC beam availability for ^225^Ac production to allow for longer implantations; and utilization of alternative implantation target materials (other than aluminum) to increase ^225^Ac product chemical purity. For example, future work to directly implant ion beams into metal-free media (e.g., ultra-pure water, buffers, dilute acids) is underway and would significantly decrease nonradioactive metal-impurities in the ^225^Ac fraction. In principal, ISOL-medical isotope production can provide radioisotopes of the highest radiochemical purity, as it relies on extraction and high-resolution mass separation of the ion beams. Using the current ISAC facilities, ISOL could theoretically produce up to 370 MBq of ^225^Ac per month (given the maximum measured ISAC beam intensities of 1.3 × 10^8^ ions/s and 1.6 × 10^8^ ions/s for ^225^Ac and ^225^Ra, respectively, and three 10-day implantations) (Robertson et al. 2018). Increasing the beam current from 10 to 100 μA would further increase yields 10x and replacing uranium carbide targets with thorium targets should increase yields by 8.39x. Given these potential improvements, maximum annual ^225^Ac production per ISOL facility (191 GBq/year) could surpass the production via ^229^Th decay (63 GBq/year) (Robertson et al. 2018). While ^225^Ac produced via ISOL has the advantage of low cost for grant-funded academic researchers, compared to other ^225^Ac suppliers, with the current facility, there are disadvantages related to the reliability, consistency, and frequency of production runs (see Table 1). In our experience, this has made it challenging to plan more involved in vivo studies. As new sources of ^225^Ac begin to emerge (11th International Symposium on Targeted Alpha Therapy (TAT11), 2019; Robertson et al. 2017), medical radionuclide production at TRIUMF’s ISAC facility will transition towards less accessible radionuclides in order to harness the main advantage of ISOL facilities for medical isotope production – the flexibility to provide quick access to a diverse range of high-purity medical radionuclides without needing to establish radionuclide-specific production infrastructure or processes (Robertson et al. 2018; dos Santos Augusto et al. 2014; Kunz et al. 2018). The limitations of ISAC-produced ^225^Ac for preclinical research are highlighted by challenges associated with the ^225^Ac-DOTA-CycMSH study: the relatively low levels of ^225^Ac isolated by the ISOL method required the entire isolated ^225^Ac fraction for one radiolabeling reaction to isolate enough purified radiotracer for one set of preclinical in vivo studies (see Additional file 1: Section S2). The larger amount of precursor needed to provide high RCY consequently resulted in a lower molar activity product, and though HPLC purification can be used to remove excess unlabeled peptide and increase molar activity, this also results in a loss of product. Preliminary ^225^Ac radiolabeling of DOTATOC (with 25 μg) was also accomplished, using low (37 kBq) or high (1.5 MBq) ^225^Ac radioactivities, and a similar trend in radiolabeling yield was observed. Moderately high radiolabeling yields (91 ± 5%, *n* = 3, with calculated molar activities between 1.8–2.0 kBq/nmol) were obtained when low activities were used, while yields decreased significantly (43 ± 14%, *n* = 3, with calculated molar activities between 25 and 50 kBq/nmol) when 1.5 MBq of ^225^Ac was used in the labeling reaction. Presumably, the added non-radioactive impurities introduced by the increased amount of ^225^Ac added to the radiolabeling solution in the high radioactivity labeling reactions hampers radiolabeling yields. In comparison, previously published reports of DOTATOC radiolabeling yields using (commercially available) ^229^Th-derived ^225^Ac were 90 ± 4% (Miederer et al. 2008) which yielded molar activities between 57 and 171 kBq/nmol.

The moderate to poor ^225^Ac radiolabeling yields of DOTATOC and DOTA-CycMSH with decreasing ligand concentration further highlights the need to develop chelating ligands that have high affinity for ^225^Ac^III^. Of the ligands reported in the ^225^ Ac radiolabeling studies section, the high radiochemical yields and favourable stabilities in serum of the two new ^225^Ac-complexes studied here, [^225^Ac(octapa)]^−^ and [^225^Ac(*CHX*octapa)]^−^, suggests that these ligands may be good candidates for incorporation into a bioconjugate for ^225^Ac targeted alpha therapy. Though in vitro human serum stability assays are a widely used and accepted assay to predict the in vivo robustness of radiometal-chelate complexes (Ramogida and Orvig 2013; Price and Orvig 2014), in vivo biodistribution studies of [^225^Ac(octapa)]^−^ and [^225^Ac(*CHX*octapa)]^−^ compared to [^225^Ac(DOTA)]^−^ or [^225^Ac(macropa)]^+^ would validate the in vitro results and assess the clearance and uptake profiles of the radiometal complexes and indeed are planned for future studies as we secure other higher sources of ^225^Ac. The results of the La^III^ exchange studies should also be interpreted with caution; this assay is helpful to assess the kinetic inertness or the kinetic off rates of the pre-formed ^225^Ac-complexes, but the use of a “surrogate element” (i.e. La^III^) introduces potential bias as it is not known if the chelators will have the same affinity for Ac^III^ as they do La^III^. For example, the [^225^Ac(*CHX*octapa)]^−^ complex outperforms [^225^Ac(macropa)]^+^ in the 7 day serum stability challenge (95.6 ± 1.6 and 90%, respectively), yet [^225^Ac(*CHX*octapa)]^−^ dissociates (3.9 ± 0.6% intact after 7 days) in the presence of excess La^III^; this may be due to (*CHX*octapa)^4−^ affinity for La^III^ and less so than the inertness of [^225^Ac(*CHX*octapa)]^−^. Additional competitive radiolabeling experiments in which excess of non-radioactive ions such as Na^I^, Ca^II^, Mg^II^, Fe^III^ followed by ^225^Ac^III^ and chelator would further corroborate the selectivity of a chelator for Ac^III^ (Deri et al. 2014).

Based on this preliminary data, macropa remains the most efficient ligand for ^225^Ac radiolabeling, though all the “pa” ligands tested represent an advantage compared to DOTA as they can complex ^225^Ac to at least some degree at ambient temperature.

The preliminary biodistribution studies of [^225^Ac]Ac-CCZ01048 revealed elevated tumour uptake of 5.23 ± 1.78%ID/g at 2 h p.i. While blocking studies confirmed that the uptake was receptor mediated, the excess amount of unlabelled precursor delayed the clearance of [^225^Ac]Ac-CCZ01048, and thus higher background levels in the kidneys and liver were observed, which was expected and in line with the results of the [^68^Ga]Ga-CCZ01048 PET imaging study (Zhang et al. 2017). The previously reported [^68^Ga]Ga-CCZ01048 tracer exhibited a higher tumour uptake of 21.9 ± 4.6%ID/g at 2 h p.i. (Zhang et al. 2017); this large discrepancy in tumour uptake may be explained by the differences in molar activity of the final injected tracers. The molar activity of the ^225^Ac-bioconjugate (200 kBq/nmol) is equivalent to one in every ~ 2440 peptide molecules labelled with ^225^Ac, while the reported molar activity of the injected ^68^Ga-tracer (236.8 ± 66.6 MBq/nmol) (Zhang et al. 2017) is equivalent to one in every ~ 433 peptide molecules labelled with ^68^Ga; a 5.6x difference. The lower radioactivity levels obtainable with ISOL derived ^225^Ac, coupled with the moderate radiolabeling yields precluded the isolation of an ^225^Ac-labelled conjugate with higher molar activity. Nonetheless, these preliminary studies show ISOL-derived ^225^Ac can be used to drive preclinical radiopharmaceutical development. Additionally, in vivo studies show proof-of-concept that CCZ01048 may be a promising candidate for further therapy studies with ^225^Ac targeted alpha therapy, which can be enabled by ^225^Ac obtained via other ^225^Ac production routes.

## Conclusions

^225^Ra/^225^Ac ion beams produced at TRIUMF’s ISOL facility, were successfully used to produce ^225^Ac quantities sufficient for preclinical radiopharmaceutical screening (up to 18.0 MBq). The radiochemical separation of ^225^Ac from ^225^Ra was achieved on a solid phase extraction resin in high yields (> 98%) and as a radionuclidically pure product. The purified ^225^Ac was in a form amenable for radiolabeling and was used to screen a library of acyclic picolinic polydentate chelators for their ability to bind ^225^Ac^III^, and perform preliminary radiolabeling of DOTA-bioconjguates. Finally, a DOTA-CycMSH conjugate was radiolabeled with ISOL-derived ^225^Ac, and proof-of-principle biodistribution studies were conducted in mice bearing B16F10 tumours. Receptor-mediated localization of the ^225^Ac-DOTA-CycMSH tracer was confirmed by a blocking experiment, suggesting that this peptide would be a promising candidate for further preclinical testing in vivo for targeted alpha therapy with ^225^Ac. In conclusion, together these studies show that ISOL-derived ^225^Ac can successfully be used to screen new chelating ligands for Ac^III^ coordination and be used to drive preclinical radiopharmaceutical development.

## Additional file

Additional file 1 A comprehensive list of ^225^Ac radiolabeling studies performed, DOTA-CycMSH radiolabeling development, detailed ICP-MS results, biodistribution tables from the [^225^Ac]Ac-DOTA-CycMSH study, and representative radio-TLC chromatorgrams of the ^225^Ac in vitro serum stability assay can be found in the Additional file 1. (DOCX 421 kb)

## Data Availability

The datasets used and/or analysed during the current study are available from the corresponding author(s) on reasonable request.
