# Supplementary material for: Evaluation of polydentate picolinic acid chelating ligands and an α-melanocyte-stimulating hormone derivative for targeted alpha therapy using ISOL-produced 225Ac
Source: EJNMMI Radiopharm Chem. 2019 Aug 6;4:21. doi: 10.1186/s41181-019-0072-5 (PMC6684685; doi:10.1186/s41181-019-0072-5)
Supplement: Supplementary file 1 — A comprehensive list of 225Ac radiolabeling studies performed, DOTA-CycMSH radiolabeling development, detailed ICP-MS results, biodistribution tables from the [225Ac]Ac-DOTA-CycMSH study, and representative radio-TLC chromatorgrams of the 225Ac in vitro serum stability assay can be found in the Additional file 1. (DOCX 421 kb) [file 41181_2019_72_MOESM1_ESM.docx]

**Supplemental**

**Evaluation of polydentate picolinic acid chelating ligands and an α-melanocyte-stimulating hormone derivative for targeted alpha therapy using ISOL-produced ^225^Ac**

Caterina F. Ramogida^1,2^*, Andrew K. H. Robertson^1,3^, Una Jermilova^1^, Chengcheng Zhang^4^, Hua Yang^1^, Peter Kunz^5^, Jens Lassen^5^, Ivica Bratanovic^1^, Victoria Brown^1^, Lily Southcott^1^, Cristina Rodríguez-Rodríguez ^3,6^, Valery Radchenko^1,9^, François Bénard^3,7,8^, Chris Orvig^9^, Paul Schaffer^1,2,8^*

^1^Life Sciences Division, TRIUMF, 4004 Wesbrook Mall, Vancouver, BC, V6T 2A3 Canada

^2^Department of Chemistry, Simon Fraser University, 8888 University Dr., Burnaby, BC, V5A 1S6 Canada

^3^Department of Physics & Astronomy, University of British Columbia, 6224 Agricultural Road, Vancouver, BC, V6T 1Z1 Canada

^4^Department of Molecular Oncology, BC Cancer Research Centre, 675 West 10^th^ Ave., Vancouver, BC, V5Z 1L3 Canada

^5^Accelerator Division, TRIUMF, 4004 Wesbrook Mall, Vancouver, BC, V6T 2A3 Canada

^6^Faculty of Pharmaceutical Sciences, University of British Columbia, 2405 Wesbrook Mall, Vancouver, BC, V6T 1Z3 Canada

^7^Department of Functional Imaging, BC Cancer, 600 West 10^th^ Ave., Vancouver, BC, V5Z 4E6 Canada

^8^Department of Radiology, University of British Columbia, 2775 Laurel St., Vancouver, BC, V5Z 1M9 Canada

^9^Medicinal Inorganic Chemistry Group, Department of Chemistry, University of British Columbia, 2036 Main Mall, Vancouver, BC, V6T 1Z1 Canada

*Correspondence: cramogida@triumf.ca; pschaffer@triumf.ca

**S1. Summary of all radiolabeling conditions evaluated**

**Table S1.** A comprehensive list of all radiolabeling parameters explored in this study, alongside previously published results for ^225^Ac ligands H_2_bispa^2^ and macropa tested with the same ^225^Ac source [1], [2].

| **Ligand** | **Molarity** | **Time** | **Temperature** | **pH** | **%RCY** | ***n*** | **Conditions to determine %RCY** |
| --- | --- | --- | --- | --- | --- | --- | --- |
| **H_6_phospa** | 1x 10^-3^ M | 1 hour | Ambient | 5.5 | 95 | 1 | Chelex column:  Used ~100-150ug Chelex resin, equilibriated with 0.15M NH_4_OAc pH=5.5 buffer. Loaded entire sample, eluted in 2mL of NH_4_OAc buffer, diluted to 20mL with MQ H_2_O and counted the samples on the gamma spectrometer |
|  | 1x 10^-4^ M | 1 hour | Ambient | 5.5 | 82 | 1 |  |
|  | 1x 10^-5^ M | 1 hour | Ambient | 5.5 | 14 | 1 |  |
|  | 1x 10^-6^ M  1x 10^-4^ M | 1 hour  1 hour | Ambient  40°C | 5.5  5.5 | 0  85 ± 1 | 1  3 |  |
| **H_4_neunpa-p-Bn- NO_2_**   | 1x 10^-3^ M | 1 hour | Ambient | 5.5 | 57 | 1 |  |
|  | 1x 10^-4^ M | 1 hour | Ambient | 5.5 | 31 | 1 |  |
|  | 1x 10^-5^ M  1x 10^-3^ M  1x 10^-4^ M | 1 hour  1 hour  1 hour | Ambient  40^o^C  40^o^C | 5.5  5.5  5.5 | 0  86 ± 4  49 ± 6 | 1  4  2 |  |
|  |  |  |  |  |  |  |  |
|  |  |  |  |  |  |  | Developed on iTLC-SA plates using 50mM EDTA buffer, pH=4 |
| **H_4_octapa** | 1x 10^-3^ M  1x 10^-4^ M | 1 hour  30 min | Ambient  Ambient | 5.5  5.5 | 97 ± 2  77 | 3  1 |  |
|  | 1x 10^-4^ M | 1 hour | Ambient | 5.5 | 97 ± 1 | 5 |  |
|  | 1x 10^-4^ M | 2 hour | Ambient | 7 | 99 | 1 |  |
|  | 1x 10^-5^ M | 1 hour | Ambient | 5.5 | 96 ± 2 | 2 |  |
|  | 1x 10^-6^ M  1x 10^-7^ M | 1 hour  1 hour | Ambient  Ambient | 5.5  5.5 | 9 ± 2  7 | 2  1 |  |
|  |  |  |  |  |  |  |  |
| **CHX-H_4_octapa**   | 1x 10^-3^ M  1x 10^-4^ M | 1 hour  30 min | Ambient  Ambient | 5.5  5.5 | 98 ± 2  90 | 4  1 |  |
|  | 1x 10^-4^ M | 1 hour | Ambient | 5.5 | 93 ± 3 | 4 |  |
|  | 1x 10^-4^ M | 2 hour | Ambient | 5.5 | 96 ± 1 | 2 |  |
|  | 1x 10^-4^ M | 2 hour | Ambient | 7 | 95 | 1 |  |
|  | 1x 10^-5^ M | 30 min | Ambient | 5.5 | 85 | 1 |  |
|  | 1x 10^-5^ M | 1 hour | Ambient | 5.5 | 93 ± 1 | 2 |  |
|  | 1x 10^-5^ M | 2 hour | Ambient | 5.5 | 97 ± 0.2 | 2 |  |
|  | 1x 10^-6^ M | 30 min | Ambient | 5.5 | 92 | 1 |  |
|  | 1x 10^-6^ M | 1 hour | Ambient | 5.5 | 93 ± 1 | 2 |  |
|  | 1x 10^-6^ M | 2 hour | Ambient | 5.5 | 97 | 1 |  |
|  | 1x 10^-7^ M | 30 min | Ambient | 5.5 | 2 | 1 |  |
|  | 1x 10^-7^ M | 1 hour | Ambient | 5.5 | 3 ± 1 | 2 |  |
|  | 1x 10^-8^ M | 30 min | Ambient | 5.5 | 2 | 1 |  |
|  | 1x 10^-8^ M | 1 hour | Ambient | 5.5 | 2 ± 0.2 | 2 |  |
| **H_2_bispa^2^ [1]**   | 1x 10^-4^ M | 1 hour | Ambient | 5.5 | 96 ± 2 | 7 | Developed on Aluminum backed silica TLC plates using 0.4M citrate buffer, pH=4 |
|  | 1x 10^-5^ M | 1 hour | Ambient | 5.5 | 94 | 1 |  |
|  | 1x 10^-6^ M  1x 10^-7^ M | 1 hour  1 hour | Ambient  Ambient | 5.5  5.5 | 64  15 | 1  1 |  |
|  | 1x 10^-8^ M | 1 hour | Ambient | 5.5 | 2 | 1 |  |
| **DOTA** | 1x 10^-3^ M | 15 min | Ambient | 5.5 | 1 | 1 | Developed on iTLC-SG plates using 0.1M NaOH/ 9% NaCl buffer |
|  | 1x 10^-3^ M | 30 min | Ambient | 5.5 | 2 | 1 |  |
|  | 1x 10^-3^ M | 1 hour | Ambient | 5.5 | 2 | 1 |  |
|  | 1x 10^-3^ M | 2 hour | Ambient | 5.5 | 11 ± 3 | 8 |  |
|  | 1x 10^-3^ M | 30 min | 85^o^C | 7 | 95 ± 4 | 5 |  |
|  | 1x 10^-3^ M | 30 min | Ambient | 7 | 7 | 1 |  |
|  | 1x 10^-4^ M | 15 min | Ambient | 5.5 | 1 ± 3 | 3 |  |
|  | 1x 10^-4^ M | 30 min | Ambient | 5.5 | 8 ± 3 | 2 |  |
|  | 1x 10^-4^ M | 1 hour | Ambient | 5.5 | 1 | 1 |  |
|  | 1x 10^-4^ M  1x 10^-5^ M  1x 10^-5^ M  1x10^7^ M | 2 hour  2 hour  30 min  30 min | Ambient  Ambient  85°C  85°C | 5.5  5.5  7  7 | 5 ± 3  0  9 ± 6  1 ± 0.4 | 5  1  3  3 |  |
| **Macropa [2]**   | (5.3 – 5.9) x 10^-5^ | 5 min | Ambient | 7 | 98 ± 2 | 6 |  |
|  | 5.4 x 10^-6^ | 5 min | Ambient | 7 | 99 | 1 | Developed on Aluminum backed silica TLC plates using 0.4M citrate buffer, pH=4 |
|  | (5.3 – 5.9) x10^-7^ | 5 min | Ambient | 7 | 98 ± 1 | 5 |  |
|  | (5.6 – 5.9) x 10^-8^ | 5 min | Ambient | 7 | 2 | 1 |  |
|  | (5.3 – 5.9) x 10^-9^ | 5 min | Ambient | 7 | 3 ± 3 | 5 |  |
|  | 5.9 x 10^-10^ | 5 min | Ambient | 7 | 3 | 1 |  |
|  |  |  |  |  |  |  |  |

**S2. [^225^Ac]Ac-CCZ01048 radiolabeling development**

The main challenges associated with the ^225^Ac-DOTA-CycMSH study was the relatively low levels of ^225^Ac radioactivity isolated by the ISOL method via either the direct implantation of ^225^Ac (~1.3 – 18.0 MBq) or via the ^225^Ra generator. Therefore, the entire isolated ^225^Ac fraction would be required for one radiolabeling reaction to isolate sufficient purified radiotracer for one set of preclinical *in vivo* studies. Initial radiolabeling studies of the DOTA-CycMSH conjugate, CCZ01048, with the ISAC ISOL derived ^225^Ac^III^ was conducted using varying amount of precursor (1 – 80 µg), with either (a) 15 µL aliquot of the ^225^Ac(NO_3_)_3_ solution, or (b) the entire ^225^Ac(NO)_3_ solution which was evaporated to dryness and reconstituted in 25 µL, results are plotted in Figure S1. Given the absolute level of non-radioactive metal impurities in the ^225^Ac solution, we anticipated the radiochemical yields to decrease as the amount of ^225^Ac solution used in a radiolabeling reaction increased. Indeed, when the entire ^225^Ac elution was used in a radiolabeling reaction, RCYs decreased significantly when the same amount of precursor was used compared to when an aliquot (15 µL of ~500 µL) of the entire ^225^Ac solution was added. Specifically, with a 15 µL ^225^Ac aliquot, RCYs were 96, 94, 87, 39, and 9% when 50, 25, 10, 5, and 1 µg of precursor was used, respectively. On the other hand, if the entire ^225^Ac elution was used in the labeling reactions, RCYs were 57, 37, and 14% with 50, 30, and 20 µg of precursor, respectively. The larger amount of precursor needed to give high RCYs consequently results in a product of lower molar activity, and though HPLC purification can be used to remove excess unlabeled peptide to increase specific activity, this process adds experimental steps and loss of product. The moderate to poor ^225^Ac radiolabeling yields of DOTA-CycMSH with decreasing ligand concentration further highlights the need to develop chelating ligands that have high affinity and specificity for ^225^Ac^III^.

**Figure S1.** ^225^Ac radiolabeling yields (RCY, %) with varying masses of precursor CCZ01048, using a 15 µL portion of the ^225^Ac elution (blue diamonds), or the entire ^225^Ac elution which was reconstituted in a total volume of 25 µL (orange circles).

**S3. Detailed ICP-MS results**

**Table S2.** Trace metal content in ppb (µg/L) determined by ICP-MS (*n* = 2)

|  | **ppb** | | | | | | | | | |
| --- | --- | --- | --- | --- | --- | --- | --- | --- | --- | --- |
|  | **Be** | **Al** | **Ca*** | **Sc** | **Ti** | **Cr** | **Mn** | **Fe** | **Co** | **Ni** |
| **Target** | 0.10 ± 0.03 | ND | 76.1 ± 0.6 | <dl | 24 ± 6 | 102 ± 74 | 156 ± 72 | 1575 ± 1355 | 0.23 ± 0.01 | 8 ± 4 |
| **Load** | 0.055 ± 0.002 | ND | 47.4 ± 0.8 | 0.07 ± 0.02 | 15 ± 1 | 46 ± 11 | 66 ± 11 | 274 ± 45 | 0.11 ± 0.02 | 18 ± 15 |
| **Wash** | 0.018 ± 0.001 | ND | 63 ± 8 | 0.061 ± 0.003 | 15 ± 1 | 11 ± 3 | 15 ± 4 | 94 ± 3 | 0.04 ± 0.01 | 76 ± 94 |
| **Elute 1** | 0.06 ± 0.02 | 7709 ± 1281 | 1392 ± 208 | 0.5 ± 0.1 | 77 ± 11 | 14.9 ± 0.3 | 4.37 ± 0.02 | 202 ± 20 | 0.10 ± 0.02 | 19 ± 6 |
| **Elute 2** | 0.07 ± 0.02 | 10175 ± 872 | 745 ± 24 | 0.9 ± 0.3 | 101 ± 8 | 62 ± 63 | 7 ± 3 | 427 ± 192 | 0.6 ± 0.7 | 37 ± 17 |

|  | **ppb (Continued)** | | | | | | | | | | |
| --- | --- | --- | --- | --- | --- | --- | --- | --- | --- | --- | --- |
|  | **Cu** | **Zn** | **Ga** | **Sr** | **Y** | **Zr** | **Nb** | **Mo** | **Sn** | **W** | **Pb** |
| **Target** | 74 ± 31 | 83 ± 23 | 8 ± 3 | 1.4 ± 0.1 | 0.3 ± 0.1 | 4.15 ± 0.01 | <dl | 1.0 ± 0.1 | 1.5 ± 0.5 | 0.9 ± 0.6 | 9 ± 2 |
| **Load** | 32 ± 11 | 57 ± 25 | 3.1 ± 0.6 | 1.13 ± | 0.19 ± 0.01 | 7.8 ± 0.7 | <dl | 0.90 ± 0.05 | 0.73 ± 0.04 | 1.0 ± 0.1 | 2.2 ± 0.6 |
| **Wash** | 12 ± 5 | 36 ± 5 | 0.86 ± 0.03 | 1.69 ± 0.02 | 0.32 ± 0.01 | 7.8 ± 0.8 | 0.022 ± 0.001 | 1.226 ± 0.004 | 0.31 ± 0.01 | 0.6 ± 0.3 | 8 ± 2 |
| **Elute 1** | 47 ± 12 | 137 ± 21 | 1.8 ± 0.5 | 20 ± 1 | 3.6 ± 0.2 | 40 ± 5 | 0.21 ± 0.04 | 5.8 ± 0.8 | 0.94 ± 0.01 | 9 ± 5 | 132 ± 27 |
| **Elute 2** | 46 ± 4 | 134 ± 23 | 2.1 ± 0.4 | 11.5 ± 0.6 | 3.6 ± 0.3 | 41 ± 5 | 0.3 ± 0.1 | 8.3 ± 1.5 | 0.9 ± 0.2 | 12 ± 6 | 19 ± 1 |

**Table S3.** Trace metal content in µg determined by ICP-MS (*n* = 2)

|  | **µg** | | | | | | | | | |
| --- | --- | --- | --- | --- | --- | --- | --- | --- | --- | --- |
|  | **Be** | **Al** | **Ca*** | **Sc (x10^-4^)** | **Ti** | **Cr** | **Mn** | **Fe** | **Co (x10^-4^)** | **Ni** |
| **Target** | 0.10 ± 0.03 | ND | 0.380 ± 0.003 | <dl | 0.12 ± 0.03 | 0.5 ± 0.4 | 0.8 ± 0.4 | 8 ± 7 | 11.4 ± 0.5 | 0.04 ± 0.02 |
| **Load** | 0.055 ± 0.002 | ND | 0.237 ± 0.004 | 4 ± 1 | 0.07 ± 0.01 | 0.23 ± 0.06 | 0.33 ± 0.06 | 1.4 ± 1.2 | 6 ± 1 | 0.09 ± 0.07 |
| **Wash** | 0.018 ± 0.001 | ND | 0.25 ± 0.03 | 2.4 ± 0.1 | 0.061 ± 0.005 | 0.04 ± 0.01 | 0.06 ± 0.02 | 0.37 ± 0.01 | 1.5 ± 0.3 | 0.3 ± 0.4 |
| **Elute 1** | 0.06 ± 0.02 | 3.9 ± 0.6 | 0.7 ± 0.1 | 2.6 ± 0.7 | 0.04 ± 0.01 | 0.0075 ± 0.0001 | (21.9 ± 0.1)x10^-4^ | 0.10 ± 0.01 | 0.5 ± 0.1 | 0.010 ± 0.003 |
| **Elute 2** | 0.07 ± 0.02 | 5.1 ± 0.4 | 0.37 ± 0.01 | 4 ± 1 | 0.050 ± 0.004 | 0.03 ± 0.03 | 0.004 ± 0.002 | 0.2 ± 0.1 | 3 ± 3 | 0.02 ± 0.01 |

|  |  | **µg Continued (x10^-4^)** | | | | | | | | | |
| --- | --- | --- | --- | --- | --- | --- | --- | --- | --- | --- | --- |
|  | **Cu** | **Zn** | **Ga** | **Sr** | **Y** | **Zr** | **Nb** | **Mo** | **Sn** | **W** | **Pb** |
| **Target** | 4000 ± 2000 | 4000 ± 1000 | 400 ± 200 | 70 ± 7 | 13 ± 3 | 207.3 ± 0.3 | <dl | 50 ± 4 | 80 ± 30 | 40 ± 30 | 400 ± 100 |
| **Load** | 1600 ± 500 | 3000 ± 1000 | 150 ± 30 | 56 ± 1 | 9.4 ± 0.6 | 390 ± 3 | <dl | 45 ± 2 | 37 ± 2 | 49 ± 7 | 110 ± 30 |
| **Wash** | 500 ± 200 | 1400 ± 200 | 34 ± 1 | 67.8 ± 0.5 | 13.0 ± 0.3 | 310 ± 3 | 0.88 ± 0.5 | 49.0 ± 2 | 12.3 ± 0.2 | 20 ± 10 | 340 ± 70 |
| **Elute 1** | 230 ± 60 | 700 ± 100 | 9 ± 3 | 101 ± 5 | 18.1 ± 0.8 | 200 ± 30 | 1.0 ± 0.2 | 29 ± 4 | 4.69 ± 0.04 | 40 ± 30 | 700 ± 100 |
| **Elute 2** | 230 ± 20 | 700 ± 100 | 11 ± 2 | 57 ± 3 | 18 ± 2 | 210 ± 20 | 1.3 ± 0.6 | 41 ± 7 | 4.5 ± 0.9 | 60 ± 30 | 94 ± 6 |
|  |  |  |  |  |  |  |  |  |  |  |  |

**S4. Biodistribution Tables for [^225^Ac]Ac-CCZ01048**

**Table S4.** Biodistribution of [^225^Ac(CCZ01048)] at 2 h post-injection. Results presented as percent injected dose (%ID ± 1 standard deviation, SD) and normalized to percent injected dose per gram of tissue (%ID/g ± 1 SD). Data measured for three energy windows: A = 60 – 120 keV; B = 180 – 260 keV, C = 400 – 480 keV.

|  | **Study 16.12.2016 (non-blocking)** | | | | **Study 29.05.2017 (block)** | | | |
| --- | --- | --- | --- | --- | --- | --- | --- | --- |
|  | M.A. = | >200 kBq/nmol | inj peptide= | <0.1-0.12 | M.A. = | 1.6 kBq/nmol | inj peptide= | 8.1-10.0 |
| **Window A:** | Average %ID | | Average %ID/g | | Average %ID | | Average %ID/g | |
| organ | A %ID | ± SD | A %ID/g | ± SD | A %ID | ± SD | A %ID/g | ± SD |
| blood | 0.22 | 0.06 | 0.25 | 0.07 | 0.29 | 0.07 | 0.34 | 0.09 |
| urine | 1.03 | 1.43 | 24.02 | 7.93 | 1.76 | 2.72 | 10.28 | 5.49 |
| feces | 0.01 | 0.01 | 0.04 | 0.08 | 0.01 | 0.01 | 0.07 | 0.08 |
| heart | 0.03 | 0.01 | 0.17 | 0.05 | 0.12 | 0.03 | 0.74 | 0.14 |
| liver | 2.20 | 0.38 | 1.34 | 0.36 | 7.64 | 1.10 | 5.49 | 0.98 |
| kidneys | 1.90 | 0.56 | 4.83 | 1.58 | 3.17 | 0.59 | 8.85 | 1.19 |
| lungs | 0.13 | 0.06 | 0.72 | 0.40 | 0.11 | 0.02 | 0.63 | 0.14 |
| small intestine | 0.16 | 0.03 | 0.25 | 0.06 | 0.82 | 0.53 | 1.19 | 0.68 |
| large intestine | 0.03 | 0.01 | 0.18 | 0.10 | 0.04 | 0.02 | 0.29 | 0.12 |
| brain | 0.02 | 0.00 | 0.04 | 0.01 | 0.01 | 0.01 | 0.01 | 0.02 |
| bladder | 0.04 | 0.05 | 1.00 | 1.04 | 0.10 | 0.18 | 2.07 | 3.77 |
| spleen | 0.05 | 0.01 | 0.46 | 0.11 | 0.07 | 0.05 | 0.86 | 0.53 |
| stomach | 0.07 | 0.01 | 0.40 | 0.10 | 0.05 | 0.01 | 0.41 | 0.14 |
| pancreas | 0.01 | 0.01 | 0.12 | 0.07 | 0.02 | 0.01 | 0.12 | 0.08 |
| bone (femur+joint) | 0.03 | 0.01 | 0.23 | 0.06 | 0.15 | 0.03 | 0.97 | 0.23 |
| thyroids | 0.01 | 0.01 | 4.18 | 4.09 | 0.00 | 0.00 | 1.28 | 2.56 |
| tail | 0.42 | 0.22 | 0.76 | 0.38 | 1.57 | 1.44 | 3.13 | 3.06 |
| tumour | 1.79 | 1.79 | 5.23 | 1.78 | 1.07 | 0.60 | 1.15 | 0.21 |
|  |  |  |  |  |  |  |  |  |
|  | **Study 16.12.2016 (non-blocking)** | | | | **Study 29.05.2017 (block)** | | | |
|  | M.A. = | >200 kBq/nmol | inj peptide= | <0.1-0.12 | M.A. = | 1.6 kBq/nmol | inj peptide= | 8.1-10.0 |
| **Window B:** | Average %ID | | Average %ID/g | | Average %ID | | Average %ID/g | |
| organ | B %ID | ± SD | B %ID/g | ± SD | B %ID | ± SD | B %ID/g | ± SD |
| blood | 0.24 | 0.06 | 0.27 | 0.07 | 0.29 | 0.05 | 0.34 | 0.08 |
| urine | 0.96 | 1.29 | 22.87 | 5.19 | 1.71 | 2.64 | 10.25 | 5.09 |
| feces | 0.01 | 0.01 | 0.07 | 0.03 | 0.05 | 0.04 | 0.44 | 0.60 |
| heart | 0.03 | 0.01 | 0.18 | 0.05 | 0.11 | 0.04 | 0.67 | 0.26 |
| liver | 2.27 | 0.40 | 1.39 | 0.37 | 7.70 | 0.97 | 5.53 | 0.91 |
| kidneys | 1.84 | 0.59 | 4.69 | 1.66 | 1.74 | 2.03 | 4.53 | 5.36 |
| lungs | 0.14 | 0.09 | 0.80 | 0.60 | 0.12 | 0.05 | 0.72 | 0.35 |
| small intestine | 0.15 | 0.03 | 0.24 | 0.07 | 3.90 | 4.66 | 5.57 | 6.24 |
| large intestine | 0.05 | 0.03 | 0.28 | 0.23 | 0.06 | 0.03 | 0.40 | 0.10 |
| brain | 0.02 | 0.01 | 0.04 | 0.02 | 0.01 | 0.01 | 0.02 | 0.02 |
| bladder | 0.04 | 0.03 | 1.08 | 0.63 | 0.10 | 0.16 | 2.40 | 3.15 |
| spleen | 0.05 | 0.01 | 0.40 | 0.07 | 0.06 | 0.03 | 0.76 | 0.34 |
| stomach | 0.04 | 0.00 | 0.24 | 0.05 | 0.10 | 0.05 | 0.76 | 0.45 |
| pancreas | 0.01 | 0.01 | 0.10 | 0.04 | 0.03 | 0.02 | 0.18 | 0.14 |
| bone (femur+joint) | 0.03 | 0.01 | 0.18 | 0.07 | 0.17 | 0.04 | 1.15 | 0.30 |
| thyroids | 0.00 | 0.01 | -0.76 | 6.56 | 0.00 | 0.00 | 0.00 | 0.00 |
| tail | 0.39 | 0.21 | 0.71 | 0.36 | 1.64 | 1.48 | 3.28 | 3.15 |
| tumour | 1.67 | 1.68 | 4.86 | 1.67 | 1.06 | 0.56 | 1.16 | 0.20 |
|  |  |  |  |  |  |  |  |  |
|  | **Study 16.12.2016 (non-blocking)** | | | | **Study 29.05.2017 (block)** | | | |
|  | M.A. = | >200 kBq/nmol | inj peptide= | <0.1-0.12 | M.A. = | 1.6 kBq/nmol | inj peptide= | 8.1-10.0 |
| **Window C:** | Average %ID | | Average %ID/g | | Average %ID | | Average %ID/g | |
| organ | C %ID | ± SD | C %ID/g | ± SD | C %ID | ± SD | C %ID/g | ± SD |
| blood | 0.19 | 0.07 | 0.22 | 0.08 | 0.15 | 0.07 | 0.18 | 0.10 |
| urine | 2.05 | 3.38 | 43.62 | 36.21 | 1.04 | 1.53 | 4.20 | 7.33 |
| feces | 0.01 | 0.04 | 0.05 | 0.26 | 0.08 | 0.04 | 0.64 | 0.55 |
| heart | 0.03 | 0.05 | 0.14 | 0.25 | 0.44 | 0.80 | 3.00 | 5.54 |
| liver | 1.98 | 0.41 | 1.22 | 0.37 | 4.39 | 1.42 | 3.17 | 1.16 |
| kidneys | 1.60 | 0.45 | 4.06 | 1.28 | 1.93 | 0.43 | 5.44 | 1.29 |
| lungs | 0.15 | 0.11 | 0.82 | 0.63 | 0.07 | 0.07 | 0.42 | 0.36 |
| small intestine | 0.15 | 0.07 | 0.23 | 0.08 | 1.55 | 1.60 | 2.22 | 2.13 |
| large intestine | 0.04 | 0.04 | 0.22 | 0.17 | 0.11 | 0.10 | 0.69 | 0.55 |
| brain | 0.02 | 0.02 | 0.04 | 0.05 | 0.02 | 0.03 | 0.05 | 0.07 |
| bladder | 0.06 | 0.02 | 2.07 | 0.92 | 0.05 | 0.09 | 1.09 | 1.89 |
| spleen | 0.06 | 0.03 | 0.54 | 0.28 | 0.06 | 0.11 | 0.70 | 1.28 |
| stomach | 0.01 | 0.03 | 0.02 | 0.17 | 0.09 | 0.09 | 0.76 | 0.82 |
| pancreas | 0.01 | 0.03 | 0.04 | 0.33 | 0.07 | 0.05 | 0.49 | 0.43 |
| bone (femur+joint) | 0.05 | 0.05 | 0.36 | 0.39 | 0.08 | 0.07 | 0.52 | 0.47 |
| thyroids | -0.01 | 0.03 | -7.77 | 19.68 | 0.02 | 0.04 | 23.42 | 37.75 |
| tail | 0.45 | 0.22 | 0.81 | 0.39 | 1.14 | 1.00 | 2.28 | 2.13 |
| tumour | 1.40 | 1.39 | 4.17 | 1.45 | 0.54 | 0.26 | 0.61 | 0.24 |

**S5. Radio-TLC chromatograms of ^225^Ac serum stability assays**

**Figure S2. Representative TLC radio-chromatograms of control serum competition assay. ‘Free’ ^225^Ac incubated with human serum and aliquot spotted on Al backed TLC plates at t = 1 day (top) and 4 days (bottom), developed using EDTA (50 mM, pH 5) mobile phase and counted the following day to ensure equilibrium, showing all ^225^Ac eluted with the solvent from (R_f_ > 0).**

**Figure S3. Representative TLC radio-chromatograms of [^225^Ac(octapa)]^-^ versus serum competition assay. Pre-formed [^225^Ac(octapa)]^-^ complex incubated with human serum and aliquot spotted on Al backed TLC plates at t = 1 hour (top) and 7 days (bottom), developed using EDTA (50 mM, pH 5) mobile phase and counted the following day to ensure equilibrium, showing [^225^Ac(octapa)]^-^ complex remains at baseline (R_f_ = 0).**

**References**

[1] P. Comba, U. Jermilova, C. Orvig, B. O. Patrick, C. F. Ramogida, K. Rück, C. Schneider, and M. Starke, “Octadentate picolinic acid-based bispidine ligand for radiometal ions,” *Chem. - A Eur. J.*, 2017, 15945-15956.

[2] N. A. Thiele, V. Brown, J. M. Kelly, A. Amor-Coarasa, U. Jermilova, S. N. MacMillan, A. Nikolopoulou, S. Ponnala, C. F. Ramogida, A. K. H. Robertson, C. Rodríguez-Rodríguez, P. Schaffer, C. Williams Jr., J. W. Babich, V. Radchenko, and J. J. Wilson, “An Eighteen-Membered Macrocyclic Ligand for Actinium-225 Targeted Alpha Therapy,” *Angew. Chemie*, vol. 56, no. 46, 2017, 14712-14717.
